# The association of multi-system health status and its longitudinal changes with cardiovascular disease risk: insights from a secondary analysis of four multinational cohort studies

**DOI:** 10.7189/jogh.16.04277

**Published:** 2026-08-28

**Authors:** Yijing Xin, Xifeng Qian, Yanmin Yang

**Affiliations:** State Key Laboratory of Cardiovascular Disease of China, National Center for Cardiovascular Diseases, National Clinical Research Center of Cardiovascular Diseases, Fuwai Hospital, Chinese Academy of Medical Sciences and Peking Union Medical College, Beijing, China

**Keywords:** cardiovascular disease, cognitive function, depression, body mass index, grip strength, longitudinal changes

## Abstract

**Background:**

Cardiovascular diseases (CVDs) are influenced by multiple interrelated health domains, including cognitive, psychological, anthropometric, and physical functioning. However, little is known about whether overall health status across these domains and their concurrent changes over time are associated with the subsequent development of CVDs. We examined the associations of baseline multi-system health status, its longitudinal trajectories, and cumulative burden with incident CVDs.

**Methods:**

We retrieved data on 32,661 adults aged ≥45 years from four population-based studies: the China Health and Retirement Longitudinal Study, the Health and Retirement Study, the English Longitudinal Study of Ageing, and the Survey of Health, Ageing and Retirement in Europe. We followed up the participants between 2006 and 2018, depending on cohort. We assessed multi-system health across four domains (cognitive function, depressive symptoms, body mass index, and grip strength), used group-based multi-trajectory modelling to identify its distinct longitudinal patterns, and applied multivariable logistic regression to evaluate its associations with incident CVDs.

**Results:**

Greater baseline multi-system impairment was associated with higher odds of incident CVDs across cohorts, particularly among individuals with multiple unhealthy systems. We identified three distinct multi-system health trajectories: favourable maintenance, intermediate decline, and accelerated decline. Participants assigned to trajectories characterised by accelerated multi-system decline generally showed higher risk of incident CVDs than those in the other two trajectories, although the magnitude and statistical significance of associations varied across cohorts. A higher cumulative burden of unhealthy systems was associated with progressively greater odds of incident CVDs, indicating a dose-response pattern.

**Conclusions:**

In our study, poorer baseline multi-system health, unfavourable longitudinal trajectories, and greater cumulative impairment were associated with higher odds of incident CVDs. These findings highlight the potential value of monitoring multiple health domains simultaneously and longitudinally to identify individuals at elevated cardiovascular risk and to support integrated healthy ageing strategies.

Cardiovascular diseases (CVDs) remain the leading cause of mortality and disability worldwide and thus impose a substantial public health burden [1,2]. Considering the rapid population ageing at the global level [3], identifying modifiable risk factors and developing effective prevention strategies is essential for promoting healthy ageing and reducing CVD risk [4]. Traditionally, CVD prevention has focused on individual risk factors, such as hypertension, dyslipidaemia, and diabetes [4–6]. However, ageing involves concurrent and interrelated changes across multiple physiological, psychological, and functional domains [7], suggesting that individual risk factors alone may not fully capture the broader health changes associated with CVD development in older adults.

Accumulating evidence suggests that cognitive impairment, depressive symptoms, abnormal body weight, and reduced muscle strength are each associated with elevated CVD risk [9–10]. Rather than contributing to CVD risk through entirely independent pathways, these conditions may represent interconnected manifestations of multisystem biological ageing [11–17]. Shared mechanisms, including chronic inflammation, neuroendocrine dysregulation, autonomic imbalance, metabolic dysfunction, and reduced physiological reserve, may simultaneously contribute to declines across these domains and contribute to the development and progression of CVDs [18–20]. Cognitive and psychological impairments may also adversely affect health behaviours and disease self-management, further increasing CVD risk. Collectively, these mechanisms suggest that a multidimensional assessment of health may allow for a more precise determination of an individual’s cardiovascular risk.

Despite growing interest in multisystem health, several gaps remain. First, previous studies have mostly focused on individual domains or single baseline assessments, overlooking the dynamic evolution of multisystem health over time [8–10]. Second, the relationship between distinct longitudinal patterns of multisystem change and subsequent CVD risk remains poorly understood. Third, existing evidence is largely derived from single-country populations, limiting the generalisability of findings across diverse settings [21–24]. Finally, it remains unclear whether a greater burden of concurrent impairment across multiple health domains is associated with incrementally higher CVD risk beyond the contribution of any single domain.

To address these gaps, we leveraged data from four large international ageing cohorts, aiming to examine the association between baseline and longitudinal multisystem health status and incident CVDs, and assess the cumulative impact of multisystem health impairment on incident CVD risk.

## METHODS

We retrieved data on participants enrolled in four international longitudinal studies: the China Health and Retirement Longitudinal Study (CHARLS), the Health and Retirement Study (HRS), the English Longitudinal Study of Ageing (ELSA), and the Survey of Health, Ageing and Retirement in Europe (SHARE). Specifically, we considered the CHARLS waves 1–4 (2011, 2013, 2015, 2018), HRS waves 8–14 (2006–2018), ELSA waves 4, 6, 8, and 9 (2008, 2012, 2016, 2018), and SHARE waves 2, 4, 5, 6 (2006–2015), which had available data on depressive symptoms, cognitive function scores, body mass index (BMI), and grip strength. Our study adheres with the Journal of Global Health’s GRABDROP guidelines [25] (Table S22 in the **Online Supplementary Document**). We included participants aged 45 years or older, and exclude those missing baseline data on depressive symptoms, cognitive function scores, BMI, grip strength, or follow-up CVDs.

### Measurements, outcomes, and covariates

We assessed multi-system health across four domains: cognitive function, psychological health, weight status, and muscle strength. Each domain was classified as impaired or non-impaired using cohort-specific validated instruments and criteria (Tables S1–6 in the **Online Supplementary Document**) [26–31]. Multi-system health status was defined according to the number of impaired domains and categorised into four levels: 0, 1, 2, and 3–4 impaired domains, with a higher level indicating a greater burden of multidomain health impairment. The primary outcome was incident CVD, defined as a new physician diagnosis of heart attack, angina, congestive heart failure, or other heart problems self-reported by participants without CVD at baseline during follow-up. The CVD status was ascertained from participants’ reports of whether a physician had diagnosed them with any of these conditions (Table S7 in the **Online Supplementary Document**).

We selected the following demographic, socioeconomic, and health-related covariates based on their availability in the four cohorts and their established relevance to CVDs in prior research [4–6]: sex, age, education (below high school, high school, and above high school), marital status (married or others), smoking status (current smoker or not), drinking status (current drinker or not), hypertension (doctor-diagnosed history), and diabetes (doctor-diagnosed history) at baseline.

### Statistical analysis

We used multiple imputation to address missing data for certain variables separately for each cohort (Table S9 in the **Online Supplementary Document**). We generated a predefined number of imputed datasets and analysed each separately, after which we combined their results using Rubin’s rules to produce valid inferences that account for the uncertainty introduced by the imputation.

We assessed the normality of continuous variables using the Shapiro–Wilk test, summarising normally distributed ones as means and standard deviations, and non-normally distributed ones as medians and intequartile ranges. We otherwise presented categorical variables as frequencies and percentages. We compared baseline characteristics across groups using independent samples *t*-tests or Mann–Whitney U tests for continuous variables, and chi-squared or Fisher’s exact tests for categorical variables. We otherwise employed unadjusted and multivariable-adjusted logistic regression models were employed to investigate the association between baseline multi-system health status and incident CVD risk.

We used group-based multi-trajectory modelling to identify distinct subgroups of individuals exhibiting similar longitudinal patterns in cognitive function, psychological condition, grip strength, and BMI (Methods S2 and Tables S15a–15c in the **Online Supplementary Document**). Joint trajectories were modelled across three repeated assessment occasions, corresponding to waves 1, 2, and 3 (2011, 2013, and 2015) in CHARLS; waves 8/9, 10/11, and 12/13 (2006/2008, 2010/2012, and 2014/2016) in HRS; waves 4, 6, and 8 (2008, 2012, and 2016) in ELSA; and waves 2, 4, and 5 (2006–2007, 2010–2011, and 2013) in SHARE (Table S2 in the **Online Supplementary Document**). Models with two to four latent classes were estimated separately in each cohort with each trajectory modelled as a quadratic (second-degree) polynomial function of time. The optimal number of trajectory classes was determined by jointly considering model fit, classification quality, trajectory adequacy, and clinical interpretability. We selected the final three-class solution because it provided the most parsimonious balance between model fit, classification quality, trajectory separation, group size, and clinical interpretability. The three trajectories were labelled favourable maintenance, intermediate decline, and accelerated decline according to their longitudinal patterns.

In addition to the baseline analysis described above, we used unadjusted and multivariable-adjusted logistic regression models to examine the associations of longitudinal multi-system health patterns with incident CVD. Specifically, we separately examined joint trajectory classes (favourable maintenance, intermediate decline, and accelerated decline) and cumulative multi-system health status in relation to the odds of incident CVD.

We conducted stratified analyses by age (<65 *vs*. ≥65 years), sex (male *vs*. female), education level (below high school, high school, above high school), marital status (married *vs*. others), current smoking status (yes *vs*. no), current alcohol use (yes *vs*. no), hypertension (yes *vs*. no), and diabetes (yes *vs*. no) to assess the consistency of the observed associations. Interaction terms between cumulative multi-system health status and each stratification variable listed above (age, sex, education, marital status, smoking status, alcohol use, hypertension, and diabetes) were tested separately to assess potential effect modification.

We performed all analyses in SPSS, version 21 (IBM, Armonk, New York, USA) and R, version 4.3.2 (R Core Team, Vienna, Austria), with the ‘gbmt’ package used for the group-based multi-trajectory modelling. A two-sided *P*-value <0.05 indicated statistical significance.

## RESULTS

Among 101,576 participants initially recruited across the four cohorts, 1,317 were excluded due to being aged <45 years or missing age, 12,765 due to having prevalent CVD, and 32,242 due to missing baseline data on the four health domains, while 22,591 were lost to follow-up. The final baseline sample included 32,661 participants (8,825 from the CHARLS, 5,768 from the HRS, 4,101 from the ELSA, and 13,967 from the SHARE). After further excluding 12,504 participants with insufficient longitudinal data, 20,157 participants were included in the joint trajectory analysis: 5,127 from the CHARLS, 4,458 from the HRS, 3,061 from the ELSA; and 7,511 from the SHARE (Figure S1 in the **Online Supplementary Document**). Across all cohorts, participants with a higher number of unhealthy systems were older, had lower education levels, and were unmarried; exhibited higher rates of current smoking and drinking, hypertension, and diabetes; and generally had higher BMI and a higher prevalence of overweight/obesity, along with significantly lower grip strength (Tables S8 and S10–12 in the **Online Supplementary Document**).

### Association between baseline multi-system health status and CVD odds

**Figure 1:**
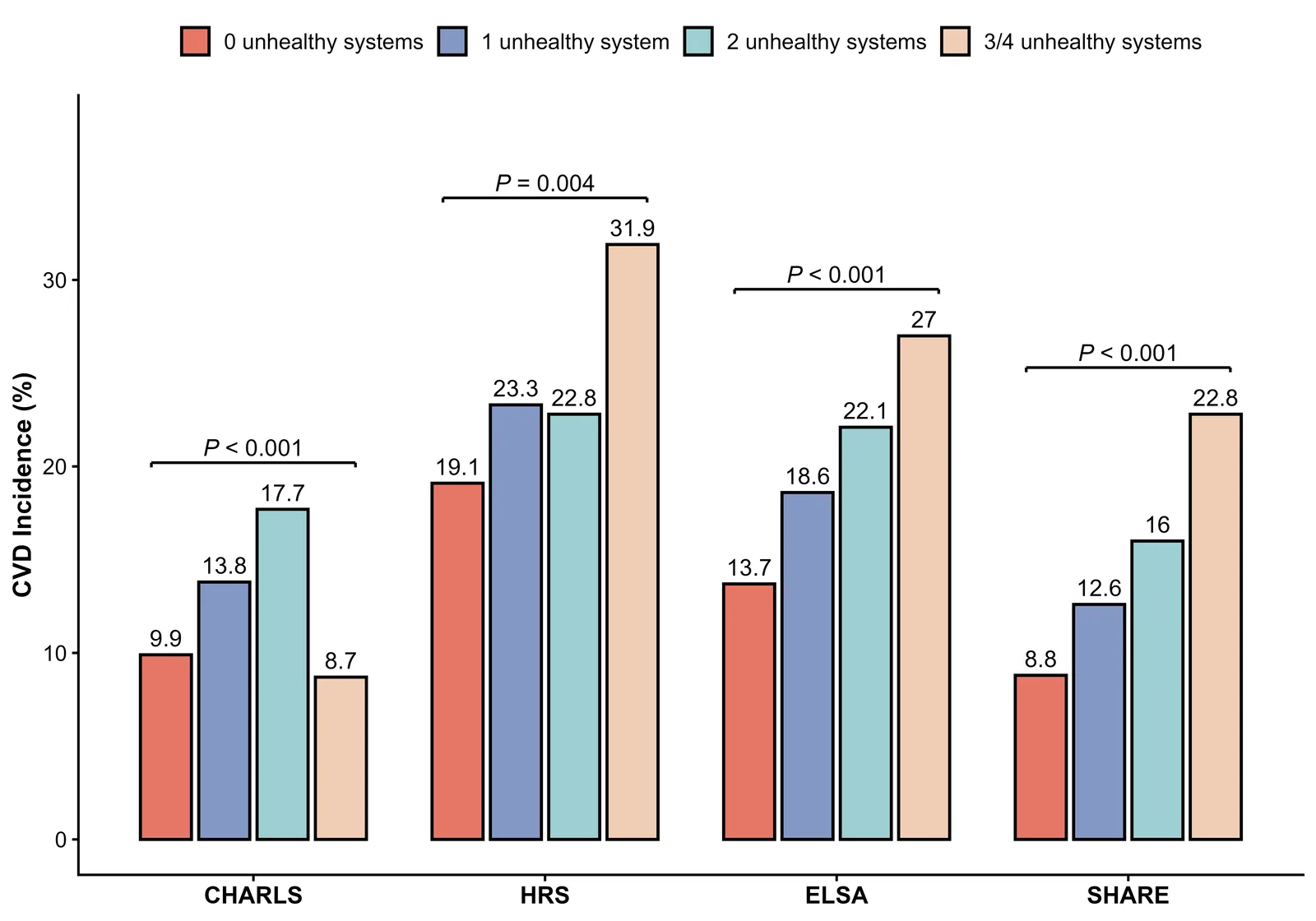
baseline multi-system health status and the incidence of CVD in the last wave. CHARLS – China Health and Retirement Longitudinal Study, CVD – cardiovascular disease, ELSA – English Longitudinal Study of Ageing, HRS – Health and Retirement Study, SHARE – Survey of Health, Ageing and Retirement in Europe.

The occurrence of incident CVD increased progressively across categories of baseline multi-system health status (0, 1, 2, and 3–4 impaired domains) (Figure 1, Table 1; Figure S3 and Tables S13 and S14 in the **Online Supplementary Document**). Participants with impairment in cognitive function, psychological health, muscle strength, or weight status also had a higher occurrence of incident CVD than their counterparts without impairment in the corresponding domain (Figure S3 in the **Online Supplementary Document**).

After multivariable adjustment (Tables S14a and S14b in the **Online Supplementary Document**), cognitive impairment was associated with higher CVD odds only in SHARE (adjusted odds ratio (aOR) = 1.239; 95% confidence interval (CI) = 1.036–1.481, *P* = 0.021). Depression was significantly associated with increased CVD odds in CHARLS (aOR = 1.476; 95% CI = 1.310–1.663, *P* < 0.001) and SHARE (aOR = 1.356; 95% CI = 1.198–1.533, *P* < 0.001). Low muscle strength was associated with elevated CVD odds in the ELSA (aOR = 1.385; 95% CI = 1.011–1.898, *P* = 0.043). Unhealthy weight status were significantly associated with increased CVD odds in the CHARLS (aOR = 1.287; 95% CI = 1.131–1.465, *P* < 0.001), ELSA (aOR = 1.323; 95% CI = 1.085–1.613, *P* = 0.006), and SHARE (aOR = 1.347; 95% CI = 1.199–1.513, *P* < 0.001).

The number of unhealthy systems exhibited a clear dose-response relationship with CVD risk (**Figure 1**, **Table 1**; Figure S3 and Tables S13 and S14 in the **Online Supplementary Document**). Compared to individuals with no unhealthy systems, those with 1, 2, or 3/4 unhealthy systems showed progressively higher adjusted odds of CVDs. Notably, having 3/4 unhealthy systems significantly increased CVD odds in all four cohorts: CHARLS (aOR = 1.690; 95% CI = 1.258–2.270, *P* = 0.001), HRS (aOR = 1.580; 95% CI = 1.007–2.480, *P* = 0.047), ELSA (aOR = 2.100; 95% CI = 1.245–3.541,*P* = 0.005), and SHARE (aOR = 2.007; 95% CI = 1.542–2.611, *P* < 0.001).

**Table 1:** Association between baseline multi-system health status and CVD odds (after multiple interpolations)*

	CHARLS (n = 8,825)		HRS (n = 5,768)		ELSA (n = 4,101)		SHARE (n = 13,967)	
	**OR (95%CI)**	***P-*value**	**OR (95%CI)**	***P-*value**	**OR (95%CI)**	***P-*value**	**OR (95%CI)**	***P-*value**
**Unhealthy system number**								
0	ref		ref		ref		ref	
1	1.313 (1.126–1.532)	0.001	1.144 (0.953–1.373)	0.148	1.300 (1.040–1.623)	0.021	1.296 (1.135–1.479)	<0.001
2	1.631 (1.358–1.960)	<0.001	1.099 (0.862–1.402)	0.442	1.568 (1.179–2.085)	0.002	1.632 (1.383–1.926)	<0.001
3/4	1.690 (1.258–2.270)	0.001	1.580 (1.007–2.480)	0.047	2.100 (1.245–3.541)	0.005	2.007 (1.542–2.611)	<0.001

CHARLS – China Health and Retirement Longitudinal Study, CI – confidence interval, CVD – cardiovascular disease, ELSA – English Longitudinal Study of Ageing, HRS – Health and Retirement Study, OR – odds ratio, ref – reference, SHARE – Survey of Health, Ageing and Retirement in Europe

*Adjusted for age, gender, education level, marital status, hypertension, diabetes, drinking status, and smoking status.

### Joint trajectories of multi-system health status

**Figure 2:**
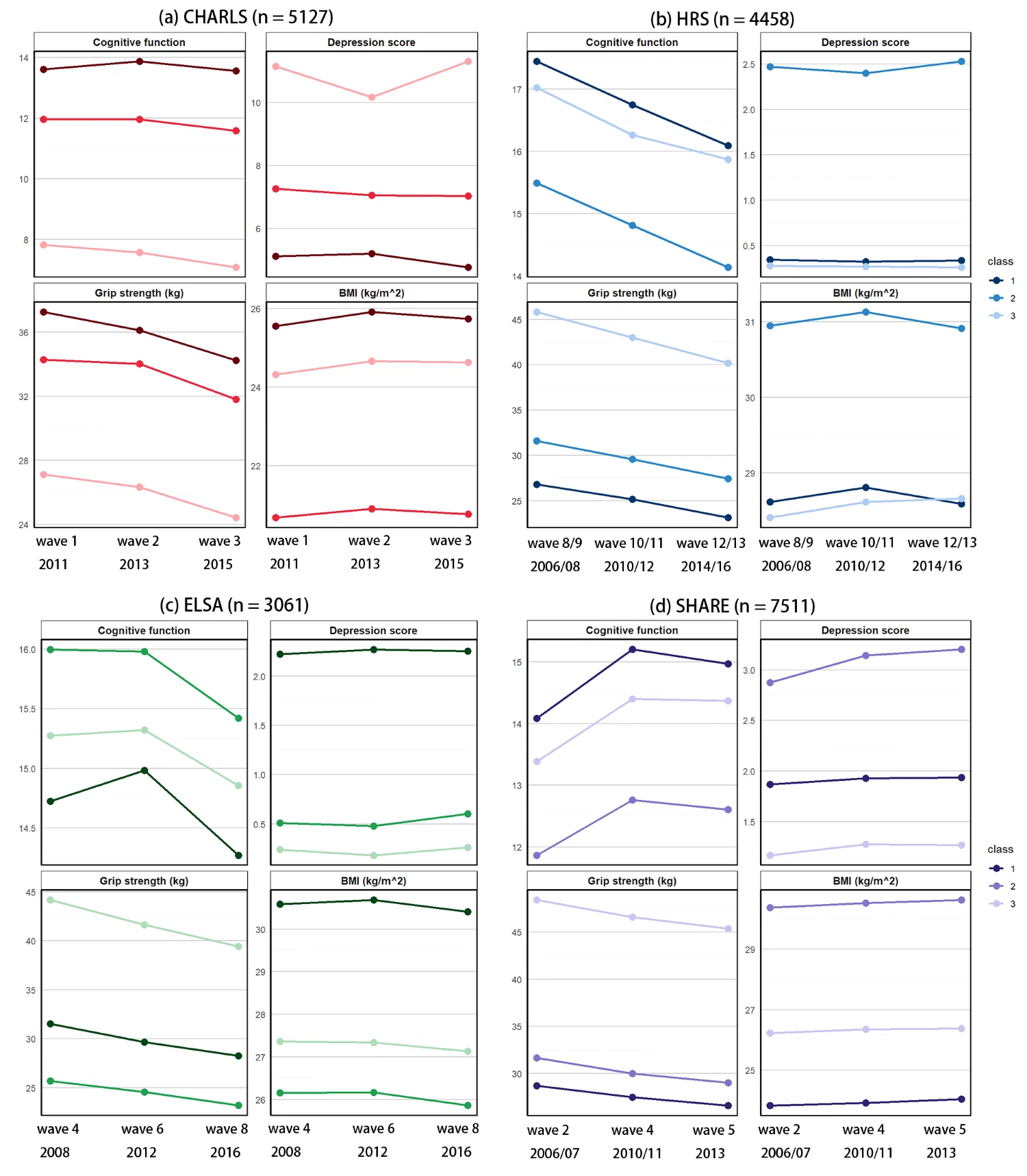
Joint trajectories of multi-system health status. Classes 1, 2, and 3 comprised 1566, 1732, and 1829 participants for CHARLS, 1735, 1559, and 1164 for HRS, 1127, 1057, and 877 for ELSA, and 2851, 2016, and 2644 for the SHARE cohort, respectively. BMI – body mass index, CHARLS – China Health and Retirement Longitudinal Study, ELSA – English Longitudinal Study of Ageing, HRS – Health and Retirement Study, SHARE – Survey of Health, Ageing and Retirement in Europe.

Group-based multi-trajectory modelling identified three latent trajectory patterns of multi-system health status that consistently emerged across all four cohorts (**Figure 2**; Figure S2 and Table S15 in the **Online Supplementary Document**). These trajectories were characterised as the favourable maintenance (model 1), intermediate decline (model 2), and accelerated decline (model 3). Model 1 characterised by a high baseline level of health indicators (cognition, grip strength, depressive symptoms, BMI) with a slow decline over time. Model 2 exhibited a moderate baseline level of health with a steady, linear decline. Model 3 defined by a low baseline level of health indicators with an accelerated decline, often accompanied by high odds for depression and abnormal/unstable BMI.

### Association between joint trajectories of multi-system health status and CVD risk

Overall, trajectory classes characterised by persistently poorer multi-system health profiles were associated with higher odds of incident CVD (**Table 2**; Tables S16a and S16b in the **Online Supplementary Document**). More specifically, in the unadjusted analyses, trajectory classes were significantly associated with CVD odds across all cohorts, although the specific patterns differed according to the cohort-specific trajectory structures. After multivariable adjustment, several associations remained significant. In CHARLS, both class 1 (aOR = 1.341; 95% confidence interval (CI) = 1.069–1.681, *P* = 0.011) and class 3 (aOR = 1.779; 95% CI = 1.589–1.993, *P* < 0.001) were associated with higher CVD odds than class 2. In HRS, class 3 was associated with increased CVD odds relative to class 2 (aOR = 1.396; 95% CI = 1.118–1.742, *P* = 0.003). We observed similar associations for class 1 in ELSA (aOR = 1.383; 95% CI = 1.089–1.756, *P* = 0.008) and class 2 in SHARE (aOR = 1.565; 95% CI = 1.296–1.889, *P* < 0.001).

**Table 2:** Association between joint trajectories of multi-system health status and CVD odds (after multiple interpolations)*

	CHARLS (n = 5,127)			HRS (n = 4,458)			ELSA (n = 3,061)			SHARE (n = 7,511)		
	**n (%)**	**OR (95%CI)**	***P*-value**	**n (%)**	**OR (95%CI)**	***P*-value**	**n (%)**	**OR (95%CI)**	***P*-value**	**n (%)**	**OR (95%CI)**	***P*-value**
**Class 1**	216 (13.8)	1.341 (1.069–1.681)	0.011	336 (19.4)	1.047 (0.920–1.191)	0.725	235 (20.9)	1.383 (1.089–1.756)	0.008	268 (9.4)	ref	
**Class 2**	164 (9.5)	ref		397 (25.5)	1.396 (1.118–1.742)	0.003	155 (14.7)	ref		346 (17.2)	1.565 (1.296–1.889)	<0.001
**Class 3**	344 (18.8)	1.779 (1.589–1.993)	<0.001	242 (20.8)	ref		166 (18.9)	1.035 (0.760–1.409)	0.829	295 (11.2)	0.942 (0.725–1.225)	0.635

CHARLS – China Health and Retirement Longitudinal Study, CI – confidence interval, CVD – cardiovascular disease, ELSA – English Longitudinal Study of Ageing, HRS – Health and Retirement Study, OR – odds ratio, ref – reference, SHARE – Survey of Health, Ageing and Retirement in Europe

*Adjusted for age, gender, education level, marital status, hypertension, diabetes, drinking status, and smoking status.

### Association between cumulative multi-system health status and CVD odds

**Figure 3:**
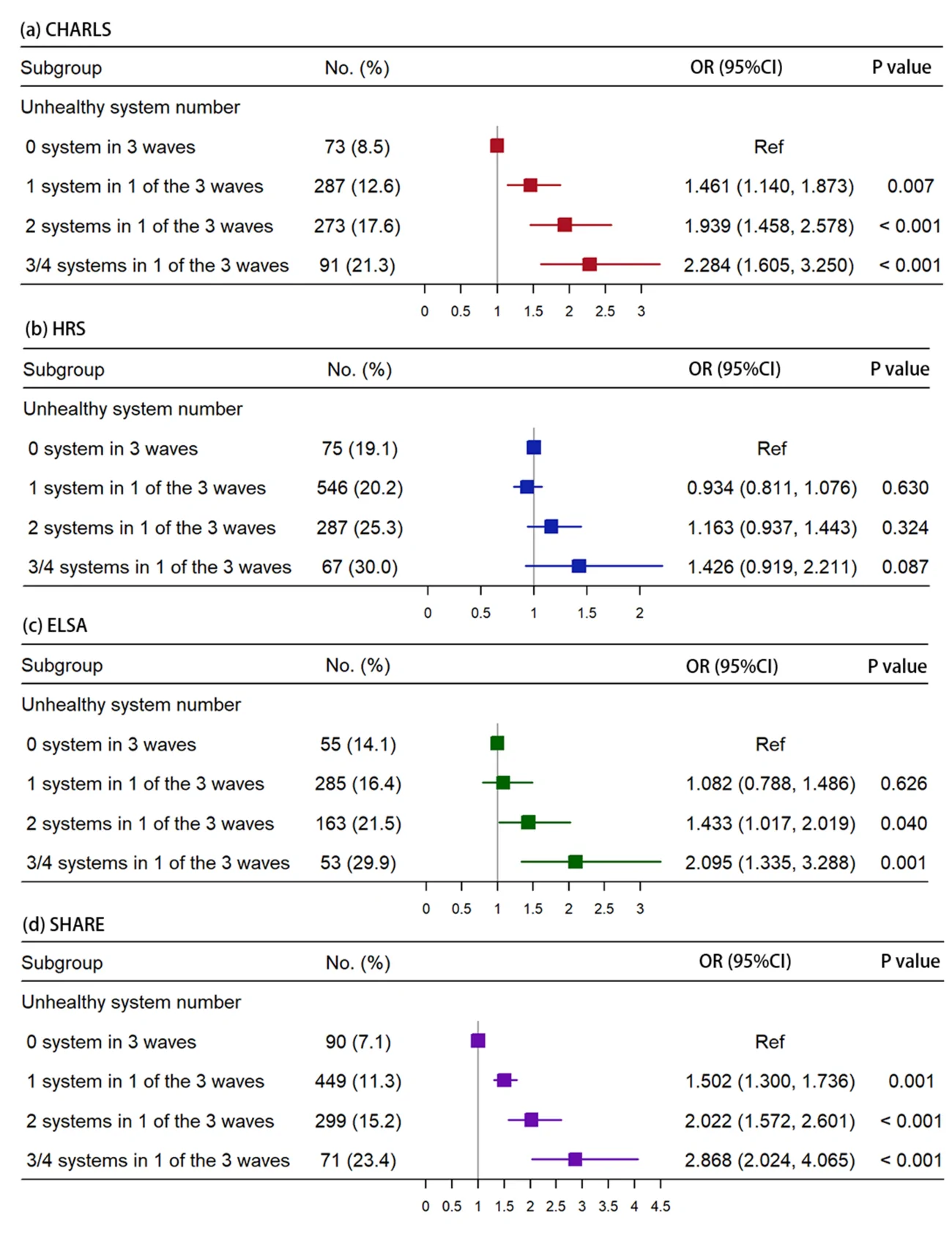
Association between cumulative multi-system health status and CVD odds (after multiple interpolations). Adjusted for age, gender, education level, marital status, hypertension, diabetes, drinking status, and smoking status. CHARLS – China Health and Retirement Longitudinal Study, CI – confidence interval, CVD – cardiovascular diseases, ELSA – English Longitudinal Study of Ageing, HRS – Health and Retirement Study, OR – odds ratio, ref – reference, SHARE – Survey of Health, Ageing and Retirement in Europe.

Greater cumulative multi-system health impairment was associated with higher odds of incident CVD, particularly in CHARLS, ELSA, and SHARE (**Figure 3**; Figures S4 and S5 and Tables S17–S19 in the **Online Supplementary Document**). In CHARLS, compared with participants with no impaired domains across all three waves, the aORs were 1.461 (95% CI = 1.140–1.873, *P* = 0.007), 1.939 (95% CI = 1.458–2.578, *P* < 0.001), and 2.284 (95% CI = 1.605–3.250, *P* < 0.001) for participants with 1, 2, and 3–4 impaired domains, respectively. Corresponding associations were observed in ELSA for 2 impaired domains (aOR = 1.433; 95% CI = 1.017–2.019, *P* = 0.040) and 3–4 impaired domains (aOR = 2.095; 95% CI = 1.335–3.288, *P* = 0.001), and in SHARE for 1 (aOR = 1.502; 95% CI = 1.300–1.736, *P* = 0.001), 2 (aOR = 2.022; 95% CI = 1.572–2.601, *P* < 0.001), and 3–4 impaired domains (aOR = 2.868; 95% CI = 2.024–4.065, *P* < 0.001). In HRS, the associations were attenuated and were not statistically significant after multivariable adjustment.

When examining individual health domains separately (Table S19 in the **Online Supplementary Document**), cumulative cognitive impairment was not consistently associated with CVD odds across cohorts. Cumulative depression showed a relatively consistent dose–response relationship with CVD odds in CHARLS, HRS, and SHARE. Associations for cumulative low muscle strength were less consistent and were mainly observed among participants with prolonged exposure. Similarly, cumulative unhealthy weight status were associated with increased CVD odds in several cohorts, particularly among individuals with persistent exposure over multiple waves.

### Subgroup analysis and sensitivity analyses

Association between the cumulative number of unhealthy systems and CVD odds remained generally consistent across subgroups (Table S20 in the **Online Supplementary Document**). Significant interactions were observed for age in SHARE (*P*-value for interaction = 0.038) and for hypertension status in CHARLS (*P*-value for interaction = 0.022) and SHARE (*P*-value for interaction <0.001). Sensitivity analyses supported the robustness of the findings (Tables S14, S16, S18, and S21 in the **Online Supplementary Document**), whereby restricting analyses to complete cases (without multiple imputation) and excluding participants who were underweight (BMI <18.5 kg/m^2^) yielded results comparable to those of the primary analysis.

## DISCUSSION

Based on data from four large international ageing cohorts, we observed that adverse multi-system health status was consistently associated with an increased risk of CVD among middle-aged and older adults. Importantly, these associations remained true for both baseline health status and for long-term trajectory patterns and cumulative exposure to health deficits, suggesting that CVD risk is influenced by both the severity and persistence of multi-system dysfunction over time.

Our observation of the dose–response relationship between the accumulation of unhealthy systems and CVD risk aligns with the growing body of evidence supporting the concept of health as an integrated and interconnected system, rather than a collection of independent physiological domains, as previous studies have linked individual factors such as obesity, depression, cognitive impairment, frailty, or sarcopenia to cardiovascular outcomes [8–10]. However, these conditions frequently coexist in older adults and may interact synergistically to accelerate cardiovascular deterioration. Our findings extend this prior research by demonstrating that the combined burden of impairments across cognitive, psychological, physical, and body composition domains provides a stronger and more consistent indicator of cardiovascular vulnerability than any single health domain alone. This, in turn, supports the multidimensional model of ageing, which propose that disease risk emerges from the cumulative interaction of deficits across multiple physiological and functional systems [7].

We also identified distinct latent trajectory patterns of multi-system health using a model-based classification approach. While previous longitudinal studies have primarily examined trajectories of single indicators, such as BMI [21,32], grip strength [22,23], and depression [24,33], our findings suggest that the pattern of change across multiple domains may provide additional prognostic information. Participants classified into trajectory patterns characterised by persistently poor health or accelerated decline consistently exhibited elevated CVD risk. These findings highlight that CVD risk is not solely determined by an individual’s current health status, but is also shaped by the direction and rate of health change over time. From a life-course perspective, accelerated multi-system decline may reflect progressive loss of physiological resilience and reduced capacity to adapt to biological and environmental stressors, ultimately increasing susceptibility to cardiovascular disease [34].

The cumulative analyses further emphasised the importance of prolonged exposure to adverse health conditions, as participants with repeated impairment across multiple survey waves generally had higher risk of CVD than those with lower cumulative exposure to impaired health domains. This finding is consistent with the concept of cumulative biological burden, whereby repeated or sustained physiological dysregulation exerts long-term detrimental effects on cardiovascular health [35–37]. Similar mechanisms have been proposed for traditional CVD risk factors, including cumulative exposure to hypertension, hyperglycaemia, obesity, and chronic inflammation [38]. Our results suggest that cumulative deficits across multiple health domains may represent an analogous process of progressive cardiovascular risk accumulation.

Depression, cognitive impairment, unhealthy body composition, and reduced muscle strength share mechanisms including chronic low-grade inflammation, oxidative stress, endothelial dysfunction, autonomic imbalance, and hypothalamic–pituitary–adrenal axis dysregulation, which may contribute to atherosclerosis, vascular ageing, and cardiometabolic dysfunction [39,40]. Furthermore, impairments in one domain may exacerbate deficits in others. For example, depression may reduce physical activity and worsen obesity [8,18,41,42], obesity may promote systemic inflammation and accelerate cognitive decline [20], and low muscle strength may contribute to frailty and reduced functional reserve [19,43]. Cognitive impairment can independently influence health behaviours and medication adherence, indirectly affecting CVD outcomes [10,44–46]. Such interactions may create a self-reinforcing cycle of multi-system deterioration that progressively increases cardiovascular vulnerability. Therefore, the observed associations likely reflect the cumulative effects of interconnected biological, behavioural, and functional pathways, rather than the influence of any single health condition.

Our findings have potential clinical and public health implications. Broader assessment of cognitive, psychological, physical, and body composition health may help identify individuals at higher CVD risk. The trajectory and cumulative analyses of multi-system health impairment further suggest that repeated assessment may provide additional information beyond single-time-point assessment. These findings support further evaluation of integrated strategies targeting multiple health domains for healthy ageing and CVD prevention. Several limitations should be considered. First, incident CVD was primarily based on self-reported physician diagnoses, which may be subject to recall bias or misclassification. However, self-reported physician-diagnosed CVD has shown acceptable validity in previous studies [47,48], and the use of harmonised definitions facilitated comparability across CHARLS, HRS, ELSA, and SHARE. Second, despite adjustment for a wide range of covariates, residual confounding from unmeasured factors cannot be excluded. Third, measurement instruments differed across cohorts. To address this, we applied cohort-specific definitions and validated thresholds, and conducted analyses separately before synthesising our findings. Nevertheless, methodological and population differences between the four cohorts may have introduced heterogeneity; therefore, our conclusions are based on the consistency of trajectory patterns and associations, rather than direct comparisons of absolute values. Fourth, although the selected three-class group-based multi-trajectory modelling solutions showed good fit and interpretability, trajectory modelling is data-driven, and alternative class structures may be plausible. The identified groups represent latent statistical classes, rather than discrete biological subtypes and should be interpreted cautiously. Finally, the observational design precludes causal inference, and the findings may not be generalisable to younger populations or settings with substantially different demographic and healthcare characteristics

## CONCLUSIONS

In our study, greater multi-system health impairment, including its accumulation and progression over time, was associated with higher CVD risk in middle-aged and older adults. These findings support a multidimensional approach to CVD risk assessment and suggest that integrated, repeated assessment of cognitive, psychological, physical, and body composition health may complement conventional risk assessment and inform strategies for healthy ageing and CVD prevention.

## Additional material


Online Supplementary Document

## Data Availability

**Data availability:** The data supporting the findings of this study are derived from the following publicly available data sets: CHARLS (https://charls.pku.edu.cn/en/index.htm), HRS (https://hrs.isr.umich.edu), ELSA (https://www.elsa-project.ac.uk), and SHARE (https://www.share-project.org).
