# Supplementary material for: The association of multi-system health status and its longitudinal changes with cardiovascular disease risk: insights from a secondary analysis of four multinational cohort studies
Source: J Glob Health. 2026 Aug 28;16:04277. doi: 10.7189/jogh.16.04277 (PMC13523042; doi:10.7189/jogh.16.04277)
Supplement: Supplementary file 1 [file jogh-16-04277-s001.pdf]

Supplement to: Xin Y, Qian X, Yang Y. The association of multi-system health status and its longitudinal changes with cardiovascular disease risk: insights from a secondary analysis of four multinational cohort studies. J Glob Health. 2026;16:04277.

Supplementary information

Table S1 The definition of multi-system health status

Table S2 The assessment time of indicators for multi-system health status

Table S3 Measurement of cognitive function in CHARLS, HRS, ELSA, and SHARE

Table S4 Measurement of depression in CHARLS, HRS, ELSA, and SHARE

Table S5 Measurement of weight status classification in CHARLS, HRS, ELSA, and SHARE

Table S6 Measurement of grip strength in CHARLS, HRS, ELSA, and SHARE

Table S7 The specific questions used to assess cardiovascular conditions in each cohort

Table S8 Comparison of baseline characteristics between the analytic and excluded participants

Table S9 Missing variables and their proportions

Table S10a. Baseline characteristics of participants from four cohorts stratified by the number of unhealthy systems without multiple interpolations

Table S10b. Baseline characteristics of participants from four cohorts stratified by the number of unhealthy systems after multiple interpolations

Table S11 Baseline characteristics of participants from four cohorts stratified by CVD in last wave without multiple interpolations

Table S12 Baseline characteristics of participants from four cohorts stratified by CVD in last wave after multiple interpolations

Table S13 Association between baseline multi-system health status and CVD odds (unadjusted)

Table S14a. Association between baseline multi-system health status and CVD odds (without multiple interpolations)

Table S14b. Association between baseline multi-system health status and CVD odds (after multiple interpolations)

Table S15a. Fit indices for 2 to 4 class GBMTM models across four cohorts

Table S15b. Entropy values for the selected 3 class models

Table S15c. Specific parameters of the multi-trajectory model

Table S16a. Association between joint trajectories of multi-system health status and CVD odds (without multiple interpolations)

Table S16b. Association between joint trajectories of multi-system health status and CVD odds (after multiple interpolations)

Table S17 Association between cumulative multi-system health status and CVD odds (unadjusted)

Table S18 Association between cumulative multi-system health status and CVD odds (without multiple interpolations)

Table S19 Association between cumulative multi-system health status and CVD odds (after multiple interpolations)

Table S20 subgroup analysis of cumulative unhealthy system number and CVD odds (after multiple interpolations)

Table S21 Association between cumulative multi-system health status and CVD odds (excluded underweight after multiple interpolations)

Table S22 Adherence to JoGH's GRABDROP guidelines items

Figure S1 flow chart of the population selection process

Figure S2 Multi-system health status trajectories models

Figure S3 baseline multi-system health status and the incidence of CVD in the last wave

Figure S4 Association between cumulative multi-system health status and CVD odds (unadjusted)

Figure S5 Association between cumulative multi-system health status and CVD odds (without multiple interpolations)

Supplementary Methods1: Multi-system health status assessment

Supplementary Methods2: Group-Based Multi-Trajectory Modeling

Table S1 The definition of multi-system health status

Multi-system health status	Definition
Cognitive function impairment	Participants' cognitive function scores < (age group-specific means - 1.5 * standard deviation of age group)
Psychological condition impairment	Participants' psychological condition scores ≥ the cut-off for defining depression
Unhealthy weight status	Underweight / Overweight / Obesity
Low muscle strength	Low grip strength

Table S2 The assessment time of indicators for multi-system health status

Surveys	CHARLS	HRS	ELSA	SHARE
**Baseline multi-system health status**				
Cognitive function	Wave 1 (2011)	Wave 8/9 (2006/2008)	Wave 4 (2008)	Wave 2 (2006-2007)
Psychological condition	Wave 1 (2011)	Wave 8/9 (2006/2008)	Wave 4 (2008)	Wave 2 (2006-2007)
Weight status	Wave 1 (2011)	Wave 8/9 (2006/2008)	Wave 4 (2008)	Wave 2 (2006-2007)
Grip strength	Wave 1 (2011)	Wave 8/9 (2006/2008)	Wave 4 (2008)	Wave 2 (2006-2007)
Follow up	Wave 2-4 (2013-2018)	Wave 10-14 (2010/2018)	Wave 6-9 (2012-2018)	Wave 4-6 (2010-2015)
**Dynamic changes of multi-system health status**				
Cognitive function	Wave 1, 2, 3 (2011, 2013, 2015)	Wave 8/9, wave 10/11, wave 12/13 (2006/2008, 2010/2012, 2014/2016)	Wave 4, 6, 8 (2008, 2012, 2016)	Wave 2, 4, 5 (2006-2007, 2010-2011, 2013)
Psychological condition	Wave 1, 2, 3 (2011, 2013, 2015)	Wave 8/9, wave 10/11, wave 12/13 (2006/2008, 2010/2012, 2014/2016)	Wave 4, 6, 8 (2008, 2012, 2016)	Wave 2, 4, 5 (2006-2007, 2010-2011, 2013)
Weight status	Wave 1, 2, 3 (2011, 2013, 2015)	Wave 8/9, wave 10/11, wave 12/13 (2006/2008, 2010/2012, 2014/2016)	Wave 4, 6, 8 (2008, 2012, 2016)	Wave 2, 4, 5 (2006-2007, 2010-2011, 2013)

Grip strength	Wave 1, 2, 3 (2011, 2013, 2015)	Wave 8/9, wave 10/11, wave 12/13 (2006/2008, 2010/2012, 2014/2016)	Wave 4, 6, 8 (2008, 2012, 2016)	Wave 2, 4, 5 (2006-2007, 2010-2011, 2013)
The end point time of observation	Wave 4 (2018)	Wave 14 (2018)	Wave 9 (2018)	Wave 6 (2015)

Abbreviations: CHARLS, China Health and Retirement Longitudinal Study; HRS, Health and Retirement Study; ELSA, English Longitudinal Study of Ageing; SHARE, Survey of Health, Ageing and Retirement in Europe.

Table S3 Measurement of cognitive function in CHARLS, HRS, ELSA, and SHARE

**Surveys**	**CHARLS**[1]	**HRS**[2, 3]	**ELSA**[2, 3]	**SHARE**
Wave	w1-w3	w1: w8/w9;<br>w2:w10/w11;<br>w3:w12/w13	w4,w6,w8	Wave 2, 4, 5
Dimensions + Scoring method	① Episodic memory (score range 0-10)<br>● Episodic memory consists of immediate word memory and delayed word memory. And interviewees were provided a 10-word list and and asked to repeat them instantly (immediate memory) and four minutes later (delayed memory).<br>② Mental intactness [Serial7's + Drawing Picture + Orientation] (score	① Episodic memory (score range 0-20)<br>● Episodic memory task consisted of immediate and delayed memory tests for 10 common nouns.The interviewees were asked to recall as many words as possible immediately after reading the list and then delayed recalling them again for about five minutes,	① Episodic memory (score range 0-20)<br>● Episodic memory tasks included language learning and memory tests in which interviewees is asked to memorize 10 common words. The scores were calculated by adding up all the target words that interviewees	① Episodic memory (score range 0-20)<br>● Episodic memory tasks included language learning and memory tests in which interviewees is asked to memorize 10 common words. The scores were calculated by adding up all the target words that interviewees remembered immediately after hearing the list (immediate recall) and after doing a series

	range 0-11);<br>● Serial7's: Interviewees need to subtract 7 from 100 five times in succession correctly (score range 0-5);<br>● Drawing Picture: Interviewees need to draw a picture of two pentagons overlapped correctly(score range 0-1);<br>Orientation: Interviewees need to recall the year, month, day, season and week of the day of the interview correctly (score range 0-5) .	during which they completed other survey questions.<br>② Executive function [Serial 7's + Backwards Counting] (score range 0-7)<br>● Serial7's: Interviewees need to subtract 7 from the previous number, beginning with 100 in five tests (score range 0-5);<br>● Backwards Counting: Interviewees was able to successfully count backwards for 10 continuous numbers from 20. 2 points if the first answer is successful, from 1 if the second answer is successful, and from 0 if the two answers are unsuccessful (score range 0-2).	remembered immediately after hearing the list (immediate recall) and after doing a series of distractions (delayed recall recall).<br>② Orientation (score range 0-4)<br>Interviewees are asked to report the day, week, month and year, respectively.	of distractions (delayed recall recall).<br>② Orientation (score range 0-5)<br>● Mathematical Ability - Calculation and Reasoning Test This test assesses digital processing, mental arithmetic and logical reasoning abilities. Test process: It includes 5 math problems with increasing difficulty.<br>Scoring rule: 1 point will be awarded for each correct answer. Score range: 0 to 5 points. (Wave 2)<br>● Serial7's: Interviewees need to subtract 7 from the previous number, beginning with 100 in five tests (score range 0-5).
Scores	Range from 0 to 21	Range from 0 to 27	Range from 0 to 24	Range from 0 to 25

Abbreviations: CHARLS, China Health and Retirement Longitudinal Study; HRS, Health and Retirement Study; ELSA, English Longitudinal Study of Ageing; SHARE, Survey of Health, Ageing and Retirement in Europe.

1. Xu T, Ye X, Lu X, Lan G, Xie M, Huang Z, et al. Association between solid cooking fuel and cognitive decline: Three nationwide cohort studies in middle-

aged and older population. Environ Int. 2023;173:107803. https://doi.org/10.1016/j.envint.2023.107803.

2. Li C, Zhu Y, Ma Y, Hua R, Zhong B, Xie W. Association of Cumulative Blood Pressure With Cognitive Decline, Dementia, and Mortality. J Am Coll Cardiol. 2022;79:1321–35. https://doi.org/10.1016/j.jacc.2022.01.045.

3. Langa KM, Llewellyn DJ, Lang IA, Weir DR, Wallace RB, Kabeto MU, et al. Cognitive health among older adults in the United States and in England. BMC Geriatr. 2009;9:23. https://doi.org/10.1186/1471-2318-9-23.

4. Andresen EM, Malmgren JA, Carter WB, Patrick DL. Screening for depression in well older adults: evaluation of a short form of the CES-D (Center for Epidemiologic Studies Depression Scale). Am J Prev Med. 1994;10:77–84.

5. Poole L, Jackowska M. The Epidemiology of Depressive Symptoms and Poor Sleep: Findings from the English Longitudinal Study of Ageing (ELSA). Int J Behav Med. 2018;25:151–61. https://doi.org/10.1007/s12529-017-9703-y.

6. Talaei M, Feng L, Barrenetxea J, Yuan J-M, Pan A, Koh W-P. Adiposity, Weight Change, and Risk of Cognitive Impairment: The Singapore Chinese Health Study. J Alzheimers Dis. 2020;74:319–29. https://doi.org/10.3233/JAD-191052.

7. Obesity: preventing and managing the global epidemic. Report of a WHO consultation. World Health Organ Tech Rep Ser. 2000;894:i–xii, 1–253.

8. Chen L-K, Woo J, Assantachai P, Auyeung T-W, Chou M-Y, Iijima K, et al. Asian Working Group for Sarcopenia: 2019 Consensus Update on Sarcopenia Diagnosis and Treatment. J Am Med Dir Assoc. 2020;21:300-307.e2. https://doi.org/10.1016/j.jamda.2019.12.012.

9. Cruz-Jentoft AJ, Bahat G, Bauer J, Boirie Y, Bruyère O, Cederholm T, et al. Sarcopenia: revised European consensus on definition and diagnosis. Age Ageing. 2019;48:16–31. https://doi.org/10.1093/ageing/afy169.

Table S4 Measurement of depression in CHARLS, HRS, ELSA, and SHARE

**Surveys**	**CHARLS**[4]	**HRS, ELSA**[5]	**SHARE**[5]
Scale	CESD-10	CES-D-8	EUROD-12
Wave	w1-w3	HRS: w1: w8/w9; w2:w10/w11;w3:w12/w13<br>ELSA: w4,w6,w8	Wave 2, 4, 5
Items	Please tell me how you have felt and behaved during the past week.<br>Q1: I was bothered by things that don't usually bother me.<br>Q2: I had trouble keeping my mind on what I was doing.<br>Q3: I felt depressed.<br>Q4: I felt everything I did was an effort.<br>Q5: I felt hopeful about the future.<br>Q6: I felt fearful.<br>Q7: My sleep was restless.<br>Q8: I was happy.<br>Q9: I felt lonely.<br>Q10: I could not get “going”.	Please tell me if each of the following was true for you much of the time during the past week.<br>Q1: I felt depressed.<br>Q2: I felt that everything I did was an effort.<br>Q3: My sleep was restless.<br>Q4: I was happy.<br>Q5: Ifelt lonely.<br>Q6: I enjoyed life.<br>Q7: I felt sad.<br>Q8: I could not get “going”.	Please tell me if each of the following wa strue for you much of the time in the last month.<br>Q1: Have you been sad or depressed?<br>Q2: Have you hoped for the future?<br>Q3: Have you felt that you would rather be dead?<br>Q4: Do you tend to blame yoursel for feel guilty about anything?<br>Q5: Have you had trouble sleeping recently?<br>Q6: Do you have any interest in things?<br>Q7: Have you been irritable recently?<br>Q8: Have you been eating more or less than usual?<br>Q9: Have you had too little energy to do the things you wanted to do?<br>Q10: Can you concentrate on a television programme, film or radio

			programme?<br>Q11: Have you enjoyed doing any activity recently?<br>Q12: Have you cried at all?
Response	Rarely or none of the time (<1 day)=0, Some or a little of the time (1-2 days)=1, Occasionally or a moderate amount of the time (3-4 days)=2, Most or all of the time (5-7 days)=3.	Yes=1, No=0	Yes=1, No=0
Scores	Range from 0 to 30	Range from 0 to 8	Range from 0 to 12
Cut-off for defining depression	A score of 10 or higher	A score of 4 or higher	A score of 4 or higher

Abbreviations: CHARLS, China Health and Retirement Longitudinal Study; HRS, Health and Retirement Study; ELSA, English Longitudinal Study of Ageing; SHARE, Survey of Health, Ageing and Retirement in Europe..

4. Andresen EM, Malmgren JA, Carter WB, Patrick DL. Screening for depression in well older adults: evaluation of a short form of the CES-D (Center for Epidemiologic Studies Depression Scale). *Am J Prev Med*. 1994;10(2):77-84.

5. Poole L, Jackowska M. The Epidemiology of Depressive Symptoms and Poor Sleep: Findings from the English Longitudinal Study of Ageing (ELSA). *Int J Behav Med*. 2018;25(2):151-161. doi:10.1007/s12529-017-9703-y

Table S5 Measurement of weight status classification in CHARLS, HRS, ELSA, and SHARE

**Surveys**	**CHARLS**[6]	**HRS**[7]	**ELSA**[7]	**SHARE**[7]
Height	✓(measured)	✓(measured)	✓(measured)	✓(self-reported)
Weight	✓(measured)	✓(measured)	✓(measured)	✓(self-reported)

Wave	w1-w3	w1: w8/w9;<br>w2:w10/w11;<br>w3:w12/w13	w4,w6,w8	W2, w4, w5
Classification criteria of BMI	Underweight (< 18.5 kg/cm^2);<br>Normal weight (>= 18.5 kg/cm^2 and <25 kg/cm^2);<br>Overweight (>= 25 kg/cm^2 and < 28 kg/cm^2);<br>Obesity (>= 28 kg/cm^2).	Underweight (< 18.5 kg/cm^2);<br>Normal weight (>= 18.5 kg/cm^2 and <25 kg/cm^2);<br>Overweight (>= 25 kg/cm^2 and < 30 kg/cm^2);<br>Obesity (>= 30 kg/cm^2)		

Abbreviations: CHARLS, China Health and Retirement Longitudinal Study; HRS, Health and Retirement Study; ELSA, English Longitudinal Study of Ageing; SHARE, Survey of Health, Ageing and Retirement in Europe; BMI, Body Mass Index.

6. Talaei M, Feng L, Barrenetxea J, Yuan JM, Pan A, Koh WP. Adiposity, Weight Change, and Risk of Cognitive Impairment: The Singapore Chinese Health Study. *J Alzheimers Dis*. 2020;74(1):319-329. doi:10.3233/JAD-191052

7. Obesity: preventing and managing the global epidemic. Report of a WHO consultation. *World Health Organ Tech Rep Ser*. 2000;894:i-xii, 1-253.

Table S6 Measurement of grip strength in CHARLS, HRS, ELSA, and SHARE

Surveys	CHARLS	HRS	ELSA	SHARE
Grip strength	✓(measured)	✓(measured)	✓(measured)	✓(measured)
Wave	w1-w3	w1: w8/w9;<br>w2:w10/w11;<br>w3:w12/w13	w4,w6,w8	W2, w4, w5

Low muscle strength	Grip strength <28 kg for male and <18 kg for female[8]	Grip strength <27 kg for male and <16 kg for female[9]

Abbreviations: CHARLS, China Health and Retirement Longitudinal Study; HRS, Health and Retirement Study; ELSA, English Longitudinal Study of Ageing; SHARE, Survey of Health, Ageing and Retirement in Europe.

8. Chen LK, Woo J, Assantachai P, et al. Asian Working Group for Sarcopenia: 2019 Consensus Update on Sarcopenia Diagnosis and Treatment. *J Am Med Dir Assoc*. 2020;21(3):300-307.e2. doi:10.1016/j.jamda.2019.12.012

9. Cruz-Jentoft AJ, Bahat G, Bauer J, et al. Sarcopenia: revised European consensus on definition and diagnosis. *Age Ageing*. 2019;48(1):16-31. doi:10.1093/ageing/afy169

Table S7 The specific questions used to assess cardiovascular conditions in each cohort

**Cohort**	Question
**CHARLS**	Have you been told by a doctor that you have been diagnosed with a heart attack, coronary heart disease, angina, congestive heart failure, or other heart problems?
	“Other heart problems” is not a precisely defined medical term, but rather a category used to encompass heart conditions not explicitly listed as heart attack, coronary artery disease, or heart failure. These “other” conditions might include arrhythmias, valvular heart disease, or other less common heart issues.

**ELSA**	Has a doctor ever told that (or had) any of the conditions on this card? 02 Angina; 03 A heart attack (including myocardial infarction or coronary thrombosis); 04 Congestive heart failure; 05 A heart murmur; 06 An abnormal heart rhythm; 95 Any other heart trouble (SPECIFY).
	“Other heart disease” is not a precisely defined medical term, and refers to any cardiovascular condition diagnosed by a doctor, excluding specific conditions like angina, heart attack, congestive heart failure, heart murmur, or abnormal heart rhythm. ELSA participants are asked about these specific conditions, and any other reported heart-related diagnoses are categorized as “other heart disease”.
**SHARE**	Has a doctor told you that you have had a heart attack, coronary heart disease, angina, congestive heart failure, or other heart problems?
	“Other heart disease” in the SHARE context is a catch-all term for a variety of heart conditions that are not specifically listed, but have been diagnosed by a physician.
**HRS**	Has a doctor told you that you have had a heart attack, coronary heart disease, angina, congestive heart failure, or other heart problems?
	“Other heart disease” in the HRS context is a catch-all term for a variety of heart conditions that are not specifically listed, but have been diagnosed by a physician.

Abbreviations: CHARLS, China Health and Retirement Longitudinal Study; HRS, Health and Retirement Study; ELSA, English Longitudinal Study of Ageing; SHARE, Survey of Health, Ageing and Retirement in Europe.

Table S8 Comparison of baseline characteristics between the analytic and excluded participants

Variables	CHARLS (n=17708)			HRS (n=19193)*			ELSA (n=11050)			SHARE (n=37132)		
	Excluded	Included	P value	Excluded	Included	P value	Excluded	Included	P value	Excluded	Included	P value
No. (%)	8883 (50.2)	8825 (49.8)		13425 (69.9)	5768 (30.1)		6949 (62.9)	4101 (37.1)		23165 (62.4)	13967 (37.6)	
Sex (%)			0.845			0.924			0.352			< 0.001
Male	4246 (47.8)	4233 (48.0)		5731 (42.7)	2467 (42.8)		3121 (44.9)	1804 (44.0)		10472 (45.2)	5921 (42.4)	
Female	4635 (52.2)	4592 (52.0)		7694 (57.3)	3301 (57.2)		3828 (55.1)	2297 (56.0)		12693 (54.8)	8046 (57.6)	
Age (years)	60.1 ± 11.6	58.0 ± 8.7	< 0.001	68.3 ± 6.5	67.7 ± 6.7	< 0.001	68.5 ± 10.3	62.6 ± 7.6	< 0.001	65.3 ± 10.8	62.2 ± 8.5	< 0.001
Education (%)			< 0.001			0.015			< 0.001			< 0.001
Below high school	7605 (85.8)	7920 (89.7)		3094 (23.1)	1221 (21.2)		2537 (39.9)	947 (24.9)		11904 (51.4)	6103 (43.7)	
High school	997 (11.2)	811 (9.2)		7702 (57.4)	3402 (59.0)		2841 (44.7)	2039 (53.6)		7113 (30.7)	4835 (34.6)	
Above high school	266 (3.0)	94 (1.1)		2623 (19.5)	1145 (19.9)		979 (15.4)	819 (21.5)		4148 (17.9)	3029 (21.7)	
Marital status (%)			< 0.001			0.037			< 0.001			< 0.001
Married	7511	7906		8773	3860		4553	2955		15623	10448	

	(84.9)	(89.6)		(65.3)	(66.9)		(65.5)	(72.1)		(68.8)	(74.9)	
Others	1339 (15.1)	919 (10.4)		4652 (34.7)	1908 (33.1)		2394 (34.5)	1146 (27.9)		7069 (31.20	3509 (25.1)	
Now smoking (%)	2043 (25.3)	2828 (32.0)	< 0.001	1803 (13.6)	802 (14.1)	0.434	1069 (15.8)	473 (11.6)	< 0.001	4342 (19.0)	2777 (2.0)	0.027
Now drinking (%)	2722 (31.2)	3045 (34.5)	< 0.001	6696 (49.9)	2880 (49.9)	0.950	4600 (86.1)	3469 (92.3)	< 0.001	9942 (43.6)	6716 (48.3)	< 0.001
Hypertension (%)	2656 (30.6)	1879 (21.4)	< 0.001	7966 (59.3)	3371 (58.4)	0.249	3073 (44.3)	1302 (31.7)	< 0.001	9460 (41.1)	4886 (35.0)	< 0.001
Diabetes (%)	647 (7.5)	415 (4.7)	< 0.001	2976 (22.2)	1278 (22.2)	1.000	764 (11.0)	245 (6.0)	< 0.001	3179 (13.8)	1297 (9.3)	< 0.001
BMI (kg/m^2)	23.4 (20.9, 26.1)	23.1 (20.9, 25.7)	0.588	29.4 ± 5.8	29.5 ± 5.9	0.661	28.3 ± 5.4	28.3 ± 5.1	0.932	26.7 ± 4.5	26.7 ± 4.3	0.004
Grip strength (kg)	30.0 (24.0, 37.8)	31.5 (25.0, 40.0)	< 0.001	30.0 (24.0, 39.0)	30.0 (25.0, 39.0)	0.022	28.0 (20.0, 37.0)	31.0 (24.0, 41.0)	< 0.001	32.0 (25.0, 42.0)	33.0 (26.0, 44.0)	< 0.001
Cognitive function score	11.5 (7.5, 14.0)	11.5 (8.0, 14.0)	0.092	16.0 (13.0, 18.0)	16.0 (13.0, 18.0)	0.014	14.0 (11.0, 16.0)	15.0 (13.0, 17.0)	< 0.001	12.0 (9.0, 15.0)	13.0 (10.0, 15.0)	< 0.001
Depression score	8.0 (4.0, 13.0)	7.0 (3.0, 12.0)	< 0.001	1.0 (0.0, 2.0)	1.0 (0.0, 2.0)	0.243	1.0 (0.0, 2.0)	0.0 (0.0, 2.0)	< 0.001	1.0 (1.0, 3.00	1.0 (0.0, 3.0)	< 0.001

* 35686 patticipants in HRS wave 8/9, after eliminating duplicates, there are 19193 patticipants remaining

Abbreviations: CHARLS, China Health and Retirement Longitudinal Study; HRS, Health and Retirement Study; ELSA, English Longitudinal Study of Ageing; SHARE, Survey of Health, Ageing and Retirement in Europe; No. (%), Number (percentage); BMI, Body Mass Index.

Table S9 Missing variables and their proportions

Variables, n (%)	CHARLS	HRS	ELSA	SHARE
Sex	0 (0)	0 (0)	0 (0)	0 (0)
Age	0 (0)	0 (0)	0 (0)	0 (0)
Education	0 (0)	2 (< 0.1)	296 (7.2)	0 (0)
Marital status	0 (0)	0 (0)	0 (0)	10 (< 0.1)
Now smoking	0 (0)	40 (0.6)	21 (0.5)	52 (0.3)
Now drinking	0 (0)	1 (< 0.1)	342 (8.3)	57 (0.4)
Hypertension	43 (0.4)	0 (0)	0 (0)	13 (< 0.1)
Diabetes	83 (0.9)	0 (0)	0 (0)	13 (< 0.1)

Abbreviations: CHARLS, China Health and Retirement Longitudinal Study; HRS, Health and Retirement Study; ELSA, English Longitudinal Study of Ageing; SHARE, Survey of Health, Ageing and Retirement in Europe.

Table S10a. Baseline characteristics of participants from four cohorts stratified by the number of unhealthy systems without multiple interpolations

Variables	CHARLS (n=8825)					HRS (n=5768)					ELSA (n=4101)					SHARE (n=13967)				
	0	1	2	3/4	P value	0	1	2	3/4	P value	0	1	2	3/4	P value	0	1	2	3/4	P value
No.	3084 (34.9)	3833 (43.4)	1552 (17.6)	356 (4.0)		996 (17.3)	3847 (66.7)	812 (14.1	113 (2.0)		876 (21.4)	2551 (62.2)	585 (14.3)	89 (2.2)		3791 (27.1	7479	2245	452 (3.2)	

								)								)	(16.1)	(16.1)		
Sex (%)					< 0.001					< 0.001					< 0.001					< 0.001
Male	1850 (60.0)	1767 (46.1)	545 (35.1)	71 (19.9)		283 (28.4)	1535 (39.3)	254 (31.3)	25 (22.1)		320 (36.5)	1218 (47.7)	233 (39.8)	33 (37.1)		1418 (37.4)	3650 (48.8)	728 (32.4)	125 (27.7)	
Female	1234 (40.0)	2066 (53.9)	1007 (64.9)	285 (80.1)		713 (71.6)	2312 (60.1)	558 (68.7)	88 (77.9)		556 (63.5)	1333 (52.3)	352 (60.2)	56 (62.9)		2373 (62.6)	3829 (51.2)	1517 (67.6)	327 (72.3)	
Age (years)	57.5 ± 8.4	57.7 ± 8.7	59.0 ± 8.9	61.2 ± 8.7	< 0.001	64.5 ± 8.9	64.2 ± 8.3	63.7 ± 8.5	63.8 ± 8.7	0.193	61.7 ± 7.4	62.6 ± 7.4	63.6 ± 8.3	63.1 ± 8.1	< 0.001	61.5 ± 8.6	62.1 ± 8.3	62.8 ± 8.7	66.1 ± 9.6	< 0.001
Education (%)					< 0.001					< 0.001					< 0.001					< 0.001
Below high school	2652 (86.0)	3440 (89.7)	1477 (95.2)	351 (98.6)		76 (7.6)	470 (12.2)	270 (33.3)	67 (59.3)		136 (16.6)	547 (23.1)	218 (40.7)	46 (56.8)		1254 (33.1)	3187 (42.6)	1306 (58.2)	356 (78.8)	
High school	388 (12.6)	348 (9.1)	70 (4.5)	5 (1.4)		569 (57.1)	2316 (60.2)	426 (52.5)	42 (37.2)		450 (54.8)	1311 (55.4)	246 (46.0)	32 (39.5)		1380 (36.4)	2725 (36.	652 (29.0)	78 (17.3)	

																	4)			
Above high school	44 (1.4)	45 (1.2)	5 (0.3)	0 90.0)		351 (35.2)	1059 (27.5)	116 (14.3)	4 (3.5)		235 (28.6)	510 (21.5)	71 (13.3)	3 (3.7)		1157 (30.5)	1567 (21.0)	287 (12.8)	18 (4.0)	
Marital status (%)					< 0.001					< 0.001					< 0.001					< 0.001
Married	2821 (91.5)	3462 (90.3)	1336 (86.1)	287 (80.6)		694 (69.7)	2752 (71.5)	455 (56.0)	51 (45.1)		633 (72.3)	1906 (74.7)	369 (63.1)	47 (52.8)		2846 (75.1)	5702 (76.3)	1596 (71.3)	304 (67.6)	
Others	263 (8.5)	371 (9.7)	216 (13.9)	69 (19.4)		302 (30.3)	1095 (28.5)	357 (44.0)	62 (54.9)		243 (27.7)	645 (25.3)	216 (36.9)	42 (47.2)		944 (24.9)	1775 (23.7)	644 (28.8)	146 (32.4)	
Now smoking (%)	1232 (39.9)	1141 (29.8)	392 (25.3)	63 (17.7)	< 0.001	145 (14.6)	414 (10.8)	133 (16.4)	24 (21.4)	< 0.001	127 (14.5)	255 (10.0)	75 (12.9)	16 (18.2)	0.001	791 (20.9)	1502 (20.2)	412 (18.4)	72 (16.1)	0.020
Now drinking (%)	1279 (41.5)	1292 (33.7)	415 (26.7)	59 (16.6)	< 0.001	641 (64.4)	2285 (59.4)	348 (42.9)	36 (31.9)	< 0.001	768 (93.8)	2195 (93.2)	441 (86.5)	65 (86.7)	< 0.001	2081 (55.1)	3691 (49.5)	832 (37.3)	112 (25.1)	< 0.001

Hypertension (%)	463 (15.1)	902 (23.6)	425 (27.5)	89 (25.1)	< 0.001	307 (30.8)	1934 (50.3)	424 (52.2)	78 (69.0)	< 0.001	173 (19.7)	856 (33.6)	232 (39.7)	41 (46.1)	< 0.001	834 (22.0)	2826 (37.8)	996 (44.4)	230 (50.9)	< 0.001
Diabetes (%)	108 (3.5)	183 (4.8)	106 (6.9)	18 (5.1)	< 0.001	54 (5.4)	577 (15.0)	169 (20.8)	35 (31.0)	< 0.001	20 (2.3)	162 (6.4)	57 (9.7)	6 (6.7)	< 0.001	157 (4.1)	705 (9.4)	332 (14.8)	103 (22.8)	< 0.001
BMI (kg/m^2)	22.1 ± 1.7	24.2 ± 3.8	24.1 ± 4.4	23.9 ± 5.3	< 0.001	22.8 ± 1.6	30.8 ± 5.3	31.5 ± 5.8	32.4 ± 5.5	< 0.001	23.0 ± 1.5	29.4 ± 4.7	30.6 ± 5.2	31.1 ± 5.5	< 0.00	22.9 ± 1.5	27.9 ± 4.0	28.7 ± 4.5	29.2 ± 4.3	< 0.001
Weight status classification (%)					< 0.001					< 0.001					< 0.001					< 0.001
Underweight	0 (0.0)	201 (5.2)	236 (15.2)	105 (6.1)		0 (0.0)	16 (0.4)	10 (1.2)	0 (0.0)		0 (0.0)	12 (0.5)	3 (0.5)	2 (2.2)		0 (0.0)	70 (0.9)	47 (2.1)	10 (2.2)	
Normal weight	3084 (100.0)	1970 (51.4)	523 (33.7)	46 (12.9)		996 (100.0)	207 (5.4)	27 (3.3)	2 (1.8)		876 (100.0)	190 (7.4)	18 (3.1)	1 (1.1)		3791 (100.0)	1134 (15.2)	175 (7.8)	19 (4.2)	
Overweight	0 (0.0)	1075 (28.0)	522 (33.6)	123 (34.6)		0 (0.0)	1738 (45.2)	341 (42.0)	41 (36.3)		0 (0.0)	1408 (55.2)	290 (49.6)	42 (47.2)		0 (0.0)	4440 (59.4)	1335 (59.5)	253 (56.0)	
Obesity	0 (0.0)	587 (15.3)	271 (17.5)	82 (23.0)		0 (0.0)	1886 (49.0)	434 (53.4)	70 (61.9)		0 (0.0)	941 (36.9)	274 (46.8)	44 (49.4)		0 (0.0)	1835 (24.	688 (30.6)	170 (37.6)	

																	5)			
Grip strength (kg)	35.0 (29.0, 42.0)	32.0 (25.5, 40.0)	27.0 (21.6, 34.0)	17.0 (14.0, 25.0)	< 0.001	29.0 (24.0, 37.5)	31.5 (25.5, 42.5)	28.0 (23.0, 36.0)	24.5 (16.0, 30.0)	< 0.001	30.0 (25.0, 39.0)	33.0 (25.0, 43.0)	26.0 (20.0, 37.0)	21.0 (12.0, 28.0)	< 0.00	32.0 (27.0, 42.0)	35.0 (28.0, 46.0)	29.0 (23.0, 37.0)	20.0 (14.0, 26.0)	< 0.001

Abbreviations: CHARLS, China Health and Retirement Longitudinal Study; HRS, Health and Retirement Study; ELSA, English Longitudinal Study of Ageing; SHARE, Survey of Health, Ageing and Retirement in Europe; No. (%), Number (percentage); BMI, Body Mass Index.

Table S10b. Baseline characteristics of participants from four cohorts stratified by the number of unhealthy systems after multiple interpolations

Variables	**CHARLS** (n=8825)					**HRS** (n=5768)					**ELSA** (n=4101)					**SHARE** (n=13967)				
	0	1	2	3/4	P value	0	1	2	3/4	P value	0	1	2	3/4	P value	0	1	2	3/4	P value
No. (%)	3084 (34.9)	3833 (43.4)	1552 (17.6)	356 (4.0)		996 (17.3)	3847 (66.7)	812 (14.1)	113 (2.0)		876 (21.4)	2551 (62.2)	585 (14.3)	89 (2.2)		3791 (27.1)	7479 (16.1)	2245 (16.1)	452 (3.2)	
Sex (%)					**< 0.001**					**< 0.001**					**< 0.001**					**< 0.001**
Male	1850 (60.0)	1767 (46.1)	545 (35.1)	71 (19.9)		283 (28.4)	1535 (39.3)	254 (31.3)	25 (22.1)		320 (36.5)	1218 (47.7)	233 (39.8)	33 (37.1)		1418 (37.4)	3650 (48.8)	728 (32.4)	125 (27.7)	
Female	1234 (40.0)	2066 (53.9)	1007 (64.9)	285 (80.1)		713 (71.6)	2312 (60.1)	558 (68.7)	88 (77.9)		556 (63.5)	1333 (52.3)	352 (60.2)	56 (62.9)		2373 (62.6)	3829 (51.2)	1517 (67.6)	327 (72.3)	

Age (years)	57.5 ± 8.4	57.7 ± 8.7	59.0 ± 8.9	61.2 ± 8.7	**< 0.001**	64.5 ± 8.9	64.2 ± 8.3	63.7 ± 8.5	63.8 ± 8.7	0.193	61.7 ± 7.4	62.6 ± 7.4	63.6 ± 8.3	63.1 ± 8.1	**< 0.001**	61.5 ± 8.6	62.1 ± 8.3	62.8 ± 8.7	66.1 ± 9.6	**< 0.001**
Education (%)					**< 0.001**					**< 0.001**					**< 0.001**					**< 0.001**
Below high school	2652 (86.0)	3440 (89.7)	1477 (95.2)	351 (98.6)		76 (7.6)	471 (12.2)	270 (33.3)	67 (59.3)		148 (16.9)	591 (23.2)	239 (40.9)	48 (53.9)		1254 (33.1)	3187 (42.6)	1306 (58.2)	356 (78.8)	
High school	388 (12.6)	348 (9.1)	70 (4.5)	5 (1.4)		569 (57.1)	2317 (60.2)	426 (52.5)	42 (37.2)		479 (54.7)	1414 (55.4)	266 (45.5)	38 (42.7)		1380 (36.4)	2725 (36.4)	652 (29.0)	78 (17.3)	
Above high school	44 (1.4)	45 (1.2)	5 (0.3)	0 90.0)		351 (35.2)	1059 (27.5)	116 (14.3)	4 (3.5)		249 (28.4)	516 (21.4)	80 (13.7)	3 (3.4)		1157 (30.5)	1567 (21.0)	287 (12.8)	18 (4.0)	
Marital status (%)					**< 0.001**					**< 0.001**					**< 0.001**					**< 0.001**
Married	2821 (91.5)	3462 (90.3)	1336 (86.1)	287 (80.6)		694 (69.7)	2752 (71.5)	455 (56.0)	51 (45.1)		633 (72.3)	1906 (74.7)	369 (63.1)	47 (52.8)		2847 (75.1)	5704 (76.3)	1599 (71.2)	306 (67.7)	
Others	263 (8.5)	371 (9.7)	216 (13.9)	69 (19.4)		302 (30.3)	1095 (28.5)	357 (44.0)	62 (54.9)		243 (27.7)	645 (25.3)	216 (36.9)	42 (47.2)		944 (24.9)	1775 (23.7)	646 (28.8)	146 (32.3)	
Now smoking (%)	1232 (39.9)	1141 (29.8)	392 (25.3)	63 (17.7)	**< 0.001**	146 (14.7)	421 (10.9)	134 (16.5)	25 (22.1)	**< 0.001**	127 (14.5)	257 (10.1)	76 (13.0)	16 (18.0)	**0.001**	792 (20.9)	1504 (20.1)	412 (18.4)	74 (16.4)	**0.024**
Now drinking (%)	1279 (41.5)	1292 (33.7)	415 (26.7)	59 (16.6)	**< 0.001**	641 (64.4)	2285 (59.4)	348 (42.9)	36 (31.9)	**< 0.001**	819 (93.5)	2370 (92.9)	510 (87.2)	79 (88.8)	**< 0.001**	2090 (55.1)	3702 (49.5)	835 (37.2)	114 (25.2)	**< 0.001**
Hypertension (%)	465 (15.1)	902 (23.6)	427 (27.5)	89 (25.0)	**< 0.001**	307 (30.8)	1934 (50.3)	424 (52.2)	78 (69.0)	**< 0.001**	173 (19.7)	856 (33.6)	232 (39.7)	41 (46.1)	**< 0.001**	834 (22.0)	2829 (37.8)	996 (44.4)	230 (50.9)	**< 0.001**
Diabetes (%)	113 (3.7)	183 (4.8)	108 (7.0)	18 (5.1)	**< 0.001**	54 (5.4)	577 (15.0)	169 (20.8)	35 (31.0)	**< 0.001**	20 (2.3)	162 (6.4)	57 (9.7)	6 (6.7)	**< 0.001**	157 (4.1)	706 (9.4)	332 (14.8)	103 (22.8)	**< 0.001**

BMI (kg/m^2)	22.1 ± 1.7	24.2 ± 3.8	24.1 ± 4.4	23.9 ± 5.3	**< 0.001**	22.8 ± 1.6	30.8 ± 5.3	31.5 ± 5.8	32.4 ± 5.5	**< 0.001**	23.0 ± 1.5	29.4 ± 4.7	30.6 ± 5.2	31.1 ± 5.5	**< 0.00**	22.9 ± 1.5	27.9 ± 4.0	28.7 ± 4.5	29.2 ± 4.3	**< 0.001**
Weight status classification (%)					**< 0.001**					**< 0.001**					**< 0.001**					**< 0.001**
Underweight	0 (0.0)	201 (5.2)	236 (15.2)	105 (6.1)		0 (0.0)	16 (0.4)	10 (1.2)	0 (0.0)		0 (0.0)	12 (0.5)	3 (0.5)	2 (2.2)		0 (0.0)	70 (0.9)	47 (2.1)	10 (2.2)	
Normal weight	3084 (100.0)	1970 (51.4)	523 (33.7)	46 (12.9)		996 (100.0)	207 (5.4)	27 (3.3)	2 (1.8)		876 (100.0)	190 (7.4)	18 (3.1)	1 (1.1)		3791 (100.0)	1134 (15.2)	175 (7.8)	19 (4.2)	
Overweight	0 (0.0)	1075 (28.0)	522 (33.6)	123 (34.6)		0 (0.0)	1738 (45.2)	341 (42.0)	41 (36.3)		0 (0.0)	1408 (55.2)	290 (49.6)	42 (47.2)		0 (0.0)	4440 (59.4)	1335 (59.5)	253 (56.0)	
Obesity	0 (0.0)	587 (15.3)	271 (17.5)	82 (23.0)		0 (0.0)	1886 (49.0)	434 (53.4)	70 (61.9)		0 (0.0)	941 (36.9)	274 (46.8)	44 (49.4)		0 (0.0)	1835 (24.5)	688 (30.6)	170 (37.6)	
Grip strength (kg)	35.0 (29.0, 42.0)	32.0 (25.5, 40.0)	27.0 (21.6, 34.0)	17.0 (14.0, 25.0)	**< 0.001**	29.0 (24.0, 37.5)	31.5 (25.5, 42.5)	28.0 (23.0, 36.0)	24.5 (16.0, 30.0)	**< 0.001**	30.0 (25.0, 39.0)	33.0 (25.0, 43.0)	26.0 (20.0, 37.0)	21.0 (12.0, 28.0)	**< 0.001**	32.0 (27.0, 42.0)	35.0 (28.0, 46.0)	29.0 (23.0, 37.0)	20.0 (14.0, 26.0)	**< 0.001**

Abbreviations: CHARLS, China Health and Retirement Longitudinal Study; HRS, Health and Retirement Study; ELSA, English Longitudinal Study of Ageing; SHARE, Survey of Health, Ageing and Retirement in Europe; No. (%), Number (percentage); BMI, Body Mass Index.

Table S11 Baseline characteristics of participants from four cohorts stratified by CVD in last wave without multiple interpolations

Variables	CHARLS (n=8825)	HRS (n=5768)	ELSA (n=4101)	SHARE (n=13967)

CVD in the last wave	No	Yes	P value	No	Yes	P value	No	Yes	P value	No	Yes	P value
No. (%)	7650 (86.7)	1175 (13.3)		4462 (77.4)	1306 (22.6)		2297 (56.0)	1804 (44.0)		12228 (87.5)	1739 (12.5)	
Sex (%)			< 0.001			< 0.001			< 0.001			< 0.001
Male	3776 (49.4)	457 (38.9)		1557 (34.9)	540 (41.3)		1422 (42.4)	382 (51.1)		5060 (41.1)	861 (49.5)	
Female	3874 (50.6)	718 (61.1)		2905 (65.1)	766 (58.7)		1931 (57.6)	366 (48.9)		7168 (58.6)	878 (50.5)	
Age (years)	57.8 ± 8.7	59.5 ± 8.5	< 0.001	63.5 ± 8.4	66.5 ± 8.0	< 0.001	62.1 ± 7.5	64.5 ± 7.6	< 0.001	61.6 ± 8.4	66.3 ± 8.6	< 0.001
Education (%)			0.164			0.012			0.692			< 0.001
Below high school	6879 (89.9)	1041 (88.6)		676 (15.2)	207 (15.8)		766 (24.6)	181 (26.2)		5197 (42.5)	906 (52.1)	
High school	695 (9.1)	116 (9.9)		2559 (57.4)	794 (60.8)		1675 (53.8)	364 (52.6)		4318 (35.3)	517 (29.7)	
Above high school	76 (1.0)	18 (1.5)		1225 (27.5)	305 (23.4)		672 (21.6)	147 (21.2)		2713 (22.2)	316 (18.2)	
Marital status (%)			0.112			0.127			0.191			0.001
Married	6869 (89.8)	1037 (88.3)		3080 (69.0)	872 (66.8)		2431 (72.5)	524 (70.1)		9204 (75.3)	1244 (71.6)	
Others	781 (10.2)	138 (11.7)		1382 (31.0)	434 (33.2)		922 (27.5)	224 (29.9)		3015 (24.7)	494 (28.4)	
Now smoking	2513	315	<	535	181	0.077	390	83 (11.2)	0.704	2441	336 (19.4)	0.542

(%)	(32.8)	(26.8)	0.001	(12.1)	(13.9)		(11.7)			(20.0)		
Now drinking (%)	2707 (35.4)	338 (28.8)	< 0.001	2590 (58.1)	720 (55.1)	0.061	2837 (92.3)	632 (92.4)	0.937	5942 (48.8)	774 (44.7)	0.001
Hypertension (%)	1452 (19.1)	427 (36.5)	< 0.001	1970 (44.2)	773 (59.2)	< 0.001	988 (29.5)	314 (42.0)	< 0.001	4034 (33.0)	852 (49.1)	< 0.001
Diabetes (%)	315 (4.2)	100 (8.6)	< 0.001	573 (12.8)	262 (20.1)		187 (5.6)	58 (7.8)	0.026	1009 (8.3)	288 (16.6)	< 0.001
BMI (kg/m^2)	23.3 ± 3.5	24.3 ± 3.9	< 0.001	29.5 ± 5.8	29.9 ± 5.9	0.017	28.4 ± 5.0	29.2 ± 5.5	< 0.001	26.5 ± 4.3	27.7 ± 4.4	< 0.001
Weight status classification (%)			< 0.001			0.054			< 0.001			< 0.001
Underweight	483 (6.3)	59 (5.0)		17 (0.4)	9 (0.7)		16 (0.5)	1 (0.1)		117 (1.0)	10 (0.6)	
Normal weight	4971 (65.0)	652 (55.5)		982 (22.0)	250 (19.1)		929 (27.7)	156 (20.9)		4635 (37.9)	484 (27.8)	
Overweight	1455 (19.0)	265 (22.6)		1640 (36.8)	480 (36.8)		1431 (42.7)	309 (41.3)		5242 (42.9)	786 (45.2)	
Obesity	741 (9.7)	199 (16.9)		1823 (40.9)	567 (43.4)		977 (29.1)	282 (30.7)		2234 (18.3)	459 (26.4)	
Grip strength (kg)	32.0 (25.0, 40.0)	30.0 (24.2, 38.5)	< 0.001	30.5 (25.0, 40.5)	30.5 (24.0, 41.0)	0.334	30.0 (24.0, 41.0)	32.0 (24.0, 43.0)	0.206	33.0 (26.0, 44.0)	33.0 (25.0, 44.0)	0.023

Abbreviations: CHARLS, China Health and Retirement Longitudinal Study; HRS, Health and Retirement Study; ELSA, English Longitudinal Study of Ageing; SHARE, Survey of Health, Ageing and Retirement in Europe; No. (%), Number (percentage); BMI, Body Mass Index.

Table S12 Baseline characteristics of participants from four cohorts stratified by CVD in last wave after multiple interpolations

Variables	CHARLS (n=8825)			HRS (n=5768)			ELSA (n=4101)			SHARE (n=13967)		
CVD in the last wave	No	Yes	P value	No	Yes	P value	No	Yes	P value	No	Yes	P value
No. (%)	7650 (86.7)	1175 (13.3)		4462 (77.4)	1306 (22.6)		2297 (56.0)	1804 (44.0)		12228 (87.5)	1739 (12.5)	
Sex (%)			< 0.001			< 0.001			< 0.001			< 0.001
Male	3776 (49.4)	457 (38.9)		1557 (34.9)	540 (41.3)		1422 (42.4)	382 (51.1)		5060 (41.1)	861 (49.5)	
Female	3874 (50.6)	718 (61.1)		2905 (65.1)	766 (58.7)		1931 (57.6)	366 (48.9)		7168 (58.6)	878 (50.5)	
Age (years)	57.8 ± 8.7	59.5 ± 8.5	< 0.001	63.5 ± 8.4	66.5 ± 8.0	< 0.001	62.1 ± 7.5	64.5 ± 7.6	< 0.001	61.6 ± 8.4	66.3 ± 8.6	< 0.001
Education (%)			0.164			0.012			0.337			< 0.001
Below high school	6879 (89.9)	1041 (88.6)		676 (15.2)	207 (15.8)		823 (24.5)	203 (27.1)		5197 (42.5)	906 (52.1)	
High school	695 (9.1)	116 (9.9)		2559 (57.4)	794 (60.8)		1807 (53.9)	390 (52.1)		4318 (35.3)	517 (29.7)	
Above high school	76 (1.0)	18 (1.5)		1225 (27.5)	305 (23.4)		723 (21.6)	155 (20.7)		2713 (22.2)	316 (18.2)	
Marital status (%)			0.112			0.127			0.191			0.001
Married	6869	1037		3080	872		2431	524		9211	1245	

	(89.8)	(88.3)		(69.0)	(66.8)		(72.5)	(70.1)		(75.3)	(71.6)	
Others	781 (10.2)	138 (11.7)		1382 (31.0)	434 (33.2)		922 (27.5)	224 (29.9)		3017 (24.7)	494 (28.4)	
Now smoking (%)	2513 (32.8)	315 (26.8)	< 0.001	535 (12.1)	181 (13.9)	0.077	393 (11.7)	83 (11.1)	0.659	2445 (20.0)	337 (19.4)	0.564
Now drinking (%)	2707 (35.4)	338 (28.8)	< 0.001	2590 (58.1)	720 (55.1)	0.061	3086 (92.0)	692 (92.5)	0.708	5965 (48.8)	776 (44.6)	0.001
Hypertension (%)	1456 (19.1)	427 (36.4)	< 0.001	1970 (44.2)	773 (59.2)	< 0.001	988 (29.5)	314 (42.0)	< 0.001	4036 (33.0)	853 (49.1)	< 0.001
Diabetes (%)	322 (4.2)	100 (8.5)	< 0.001	573 (12.8)	262 (20.1)		187 (5.6)	58 (7.8)	0.026	1009 (8.3)	289 (16.6)	< 0.001
BMI (kg/m^2)	23.3 ± 3.5	24.3 ± 3.9	< 0.001	29.5 ± 5.8	29.9 ± 5.9	0.017	28.4 ± 5.0	29.2 ± 5.5	< 0.001	26.5 ± 4.3	27.7 ± 4.4	< 0.001
Weight status classification (%)			< 0.001			0.054			< 0.001			< 0.001
Underweight	483 (6.3)	59 (5.0)		17 (0.4)	9 (0.7)		16 (0.5)	1 (0.1)		117 (1.0)	10 (0.6)	
Normal weight	4971 (65.0)	652 (55.5)		982 (22.0)	250 (19.1)		929 (27.7)	156 (20.9)		4635 (37.9)	484 (27.8)	
Overweight	1455 (19.0)	265 (22.6)		1640 (36.8)	480 (36.8)		1431 (42.7)	309 (41.3)		5242 (42.9)	786 (45.2)	
Obesity	741 (9.7)	199 (16.9)		1823 (40.9)	567 (43.4)		977 (29.1)	282 (30.7)		2234 (18.3)	459 (26.4)	
Grip strength (kg)	32.0 (25.0,	30.0 (24.2,	< 0.001	30.5 (25.0,	30.5 (24.0,	0.334	30.0 (24.0,	32.0 (24.0,	0.206	33.0 (26.0,	33.0 (25.0, 44.0)	0.023

40.0) 38.5) 40.5) 41.0) 41.0) 43.0) 44.0)

Abbreviations: CHARLS, China Health and Retirement Longitudinal Study; HRS, Health and Retirement Study; ELSA, English Longitudinal Study of Ageing; SHARE, Survey of Health, Ageing and Retirement in Europe; No. (%), Number (percentage); BMI, Body Mass Index.

Table S13 Association between baseline multi-system health status and CVD odds (unadjusted)

	CHARLS (n=8825)		HRS (n=5768)		ELSA (n=4101)		SHARE (n=13967)	
	OR (95%CI)	P value	OR (95%CI)	P value	OR (95%CI)	P value	OR (95%CI)	P value
Cognitive function impairment								
No	Ref		Ref		Ref		Ref	
Yes	0.937 (0.761, 1.153)	0.540	0.999 (0.802, 1.245)	0.993	1.267 (0.958, 1.675)	0.097	1.441 (1.212, 1.713)	< 0.001
Depression								
No	Ref		Ref		Ref		Ref	
Yes	1.550 (1.368, 1.756)	< 0.001	1.115 (0.917, 1.356)	0.276	1.132 (0.880, 1.457)	0.334	1.363 (1.214, 1.530)	< 0.001
Low muscle strength								
No	Ref		Ref		Ref		Ref	
Yes	1.187 (0.978, 1.440)	0.083	1.680 (1.217, 2.319)	0.002	1.642 (1.212, 2.224)	0.001	1.848 (1.490, 2.293)	< 0.001
Unhealthy weight status								
No	Ref		Ref		Ref		Ref	
Yes	1.488 (1.314,	< 0.001	1.192 (1.021,	0.026	1.454 (1.200,	< 0.001	1.583 (1.416,	< 0.001

	CHARLS		HRS		ELSA		SHARE	
	1.685)		1.392)		1.762)		1.769)	
Unhealthy system number								
0	Ref		Ref		Ref		Ref	
1	1.459 (1.256, 1.694)	< 0.001	1.286 (1.080, 1.532)	0.005	1.441 (1.160, 1.791)	0.001	1.505 (1.319, 1.717)	< 0.001
2	1.953 (1.638, 2.330)	< 0.001	1.252 (0.997, 1.572)	0.053	1.782 (1.355, 2.345)	< 0.001	1.990 (1.697, 2.333)	< 0.001
3/4	2.112 (1.579, 2.825)	< 0.001	1.983 (1.295, 3.037)	0.002	2.326 (1.402, 3.859)	0.001	3.075 (2.402, 3.936)	< 0.001

Abbreviations: CHARLS, China Health and Retirement Longitudinal Study; HRS, Health and Retirement Study; ELSA, English Longitudinal Study of Ageing; SHARE, Survey of Health, Ageing and Retirement in Europe.

Table S14a. Association between baseline multi-system health status and CVD odds (without multiple interpolations)

	CHARLS (n=8825)		HRS (n=5768)		ELSA (n=4101)		SHARE (n=13967)	
	OR (95%CI)	P value	OR (95%CI)	P value	OR (95%CI)	P value	OR (95%CI)	P value
Cognitive function impairment								
No	Ref		Ref		Ref		Ref	
Yes	0.879 (0.708, 1.092)	0.244	0.892 (0.703, 1.131)	0.344	1.261 (0.911, 1.746)	0.162	1.245 (1.037, 1.495)	0.019
Depression								
No	Ref		Ref		Ref		Ref	
Yes	1.467 (1.288,	< 0.001	1.183 (0.962,	0.111	1.395 (1.037,	0.028	1.369 (1.210,	< 0.001

	1.670)		1.455)		1.876)		1.549)	
Low muscle strength								
No	Ref		Ref		Ref		Ref	
Yes	1.032 (0.841, 1.266)	0.761	1.289 (0.923, 1.801)	0.137	1.340 (0.941, 1.907)	0.105	1.089 (0.866, 1.369)	0.467
Unhealthy weight status								
No	Ref		Ref		Ref		Ref	
Yes	1.284 (1.128, 1.463)	< 0.001	1.074 (0.912, 1.265)	0.394	1.350 (1.087, 1.676)	0.007	1.342 (1.193, 1.509)	< 0.001
Unhealthy system number								
0	Ref		Ref		Ref		Ref	
1	1.310 (1.123, 1.529)	0.001	1.146 (0.854, 1.375)	0.145	1.398 (1.097, 1.782)	0.007	1.286 (1.122, 1.475)	< 0.001
2	1.616 (1.344, 1.944)	< 0.001	1.104 (0.867, 1.406)	0.423	1.732 (1.263, 2.376)	0.001	1.645 (1.390, 1.947)	< 0.001
3/4	1.673 (1.235, 2.266)	0.001	1.544 (0.981, 2.431)	0.061	2.369 (1.304, 4.304)	0.005	2.028 (1.556, 2.642)	< 0.001

Adjusted for age, gender, education level, marital status, hypertension, diabetes, drinking status, and smoking status.

Abbreviations: CHARLS, China Health and Retirement Longitudinal Study; HRS, Health and Retirement Study; ELSA, English Longitudinal Study of Ageing; SHARE, Survey of Health, Ageing and Retirement in Europe; OR, odds ratio; CI: confidence interval; Ref, reference group.

Table S14b. Association between baseline multi-system health status and CVD odds (after multiple interpolations)

	**CHARLS** (n=8825)		**HRS** (n=5768)		**ELSA** (n=4101)		**SHARE** (n=13967)	
	OR (95%CI)	P value	OR (95%CI)	P value	OR (95%CI)	P value	OR (95%CI)	P value
**Cognitive function impairment**								
No	Ref		Ref		Ref		Ref	
Yes	0.899 (0.727, 1.111)	0.329	0.887 (0.700, 1.125)	0.324	1.183 (0.886, 1.581)	0.255	1.239 (1.036, 1.481)	**0.021**
**Depression**								
No	Ref		Ref		Ref		Ref	
Yes	1.476 (1.310, 1.663)	**< 0.001**	1.188 (0.968, 1.458)	0.101	1.208 (0.929, 1.572)	0.159	1.356 (1.198, 1.533)	**< 0.001**
**Low muscle strength**								
No	Ref		Ref		Ref		Ref	
Yes	1.024 (0.923, 1.136)	0.819	1.306 (0.938, 1.818)	0.116	1.385 (1.011, 1.898)	**0.043**	1.067 (0.850, 1.340)	0.576
**Unhealthy weight status**								
No	Ref		Ref		Ref		Ref	
Yes	1.287 (1.131, 1.465)	**< 0.001**	1.073 (0.910, 1.266)	0.396	1.323 (1.085, 1.613)	**0.006**	1.347 (1.199, 1.513)	**< 0.001**

Adjusted for age, gender, education level, marital status, hypertension, diabetes, drinking status, and smoking status.

Abbreviations: CHARLS, China Health and Retirement Longitudinal Study; HRS, Health and Retirement Study; ELSA, English Longitudinal Study of Ageing; SHARE, Survey of Health, Ageing and Retirement in Europe; OR, odds ratio; CI: confidence interval; Ref, reference group.

Table S15a. Fit indices for 2-to 4-class GBMTM models across four cohorts

Cohort	Number of classes	AIC	BIC	CAIC	SSBIC
CHARLS	2-class	347035.5	347376.0	347421.0	347233.0
	3-class	342405.2	342912.1	342979.1	342699.2
	4-class	339149.3	339830.2	339920.2	339544.2
HRS	2-class	249337.6	249601.4	249637.4	249487.0
	3-class	244120.1	244537.9	244594.9	244356.7
	4-class	200206.3	200733.9	200805.9	200505.1
ELSA	2-class	170993.1	171258.2	171296.2	171137.5
	3-class	167484.7	167896.2	167955.2	167708.8

Cohort	Number of classes	AIC	BIC	CAIC	SSBIC
	4-class	165332.9	165904.9	165986.9	165644.3
SHARE	2-class	457882.5	458207.8	458248.8	458077.5
	3-class	449781.7	450273.6	450335.6	450076.6
	4-class	445719.0	446393.4	446478.4	446123.2

Table S15b. Entropy values for the selected 3-class models

Cohort	Entropy R^2
CHARLS	0.873
HRS	0.891
ELSA	0.885

Cohort	Entropy R^2
SHARE	0.879

Table S15c. Specific parameters of the multi-trajectory model

Suevey	CHARLS	HRS	ELSA	SHARE
**AIC**	369111.8	291395.3	195308.0	492833.3
**BIC**	369616.1	291896.8	195728.4	493338.7
**Avepp**				
Class 1	0.929	0.951	0.961	0.943
Class 2	0.921	0.969	0.952	0.931
Class 3	0.939	0.954	0.958	0.967
**OCC**				
Class 1	29.796	31.227	42.111	27.464
Class 2	23.047	5.707	37.359	35.892
Class 3	27.365	59.913	56.850	53.859
**Proportion of sample in each class**				
Class 1	0.305	0.389	0.368	0.380
Class 2	0.338	0.350	0.345	0.268
Class 3	0.357	0.261	0.287	0.352

Abbreviations: CHARLS, China Health and Retirement Longitudinal Study; HRS, Health and Retirement Study; ELSA, English Longitudinal Study of Ageing; SHARE, Survey of Health, Ageing and Retirement in Europe; AIC, akaike information criterion; BIC, bayesian information criterion; Avepp, average posterior

probability of class membership; OCC, odds of correct classification.

Table S16a. Association between joint trajectories of multi-system health status and CVD odds (without multiple interpolations)

	CHARLS (n=5127)			HRS (n=4458)			ELSA (n=3061)			SHARE (n=7511)		
	No. (%)	OR (95%CI)	P value	No. (%)	OR (95%CI)	P value	No. (%)	OR (95%CI)	P value	No. (%)	OR (95%CI)	P value
Class 1	216 (13.8)	1.340 (1.067, 1.681)	0.012	336 (19.4)	1.405 (0.811, 1.348)		235 (20.9)	1.367 (1.051, 1.779)	0.020	268 (9.4)	Ref	
Class 2	164 (9.5)	Ref		397 (25.5)	1.400 (1.120, 1.750)	0.003	155 (14.7)	Ref		346 (17.2)	1.568 (1.298, 1.894)	< 0.001
Class 3	344 (18.8)	1.783 (1.428, 2.228)	< 0.001	242 (20.8)	Ref	0.733	166 (18.9)	1.086 (0.779, 1.514)	0.628	295 (11.2)	0.942 (0.737, 1.205)	0.635

Adjusted for age, gender, education level, marital status, hypertension, diabetes, drinking status, and smoking status.

Abbreviations: CHARLS, China Health and Retirement Longitudinal Study; HRS, Health and Retirement Study; ELSA, English Longitudinal Study of Ageing; SHARE, Survey of Health, Ageing and Retirement in Europe; No. (%), Number (percentage); OR, odds ratio; CI: confidence interval; Ref, reference group.

Table S16b. Association between joint trajectories of multi-system health status and CVD odds (after multiple interpolations)

**CHARLS** (n=5127)	**HRS** (n=4458)	**ELSA** (n=3061)	**SHARE** (n=7511)

	No. (%)	OR (95%CI)	P value	No. (%)	OR (95%CI)	P value	No. (%)	OR (95%CI)		No. (%)	OR (95%CI)	P value
Class 1	216 (13.8)	**1.530 (1.233, 1.898)**	**< 0.001**	336 (19.4)	0.915 (0.761, 1.101)	0.347	235 (20.9)	**1.533 (1.227, 1.916)**	**< 0.001**	268 (9.4)	Ref	
Class 2	164 (9.5)	Ref		397 (25.5)	**1.302 (1.085, 1.561)**	**< 0.001**	155 (14.7)	Ref		346 (17.2)	**1.997 (1.683, 2.369)**	**< 0.001**
Class 3	344 (18.8)	**2.215 (1.815, 2.703)**	**< 0.001**	242 (20.8)	Ref		166 (18.9)	**1.359 (1.069, 1.727)**	**0.012**	295 (11.2)	**1.210 (1.017, 1.441)**	**0.032**

Unadjusted

Abbreviations: CHARLS, China Health and Retirement Longitudinal Study; HRS, Health and Retirement Study; ELSA, English Longitudinal Study of Ageing; SHARE, Survey of Health, Ageing and Retirement in Europe; No. (%), Number (percentage); OR, odds ratio; CI: confidence interval; Ref, reference group.

Table S17 Association between cumulative multi-system health status and CVD odds (unadjusted)

	**CHARLS**			**HRS**			**ELSA**			**SHARE**		
	No. (%)	OR (95%CI)	P value	No. (%)	OR (95%CI)	P value	No. (%)	OR (95%CI)	P value	No. (%)	OR (95%CI)	P value
**Cognitive function impairment**												
0 wave	592 (14.1)	Ref		825 (22.0)	Ref		451 (17.5)	Ref		814 (11.8)	Ref	

1 wave	76 (13.7)	0.968 (0.748, 1.251)	0.802	85 (21.3)	0.958 (0.745, 1.232)	0.738	72 (20.2)	1.196 (0.906, 1.579)	0.206	75 (16.2)	1.444 (1.116, 1.869)	0.005
2 waves	41 (16.8)	1.229 (0.869, 1.739)	0.243	42 (22.5)	1.028 (0.723, 1.462)	0.876	25 (24.3)	1.512 (0.953, 2.400)	0.079	15 (14.6)	1.277 (0.735, 2.218)	0.386
3 waves	15 (11.2)	0.767 (0.445, 1.322)	0.340	23 (19.7)	0.869 (0.547, 1.379)	0.551	8 (34.8)	2.516 (1.061, 5.971)	0.036	5 (15.2)	1.338 (0.515, 3.474)	0.550
**Depression**												
0 wave	255 (11.1)	Ref		749 (20.7)	Ref		424 (17.2)	Ref		498 (10.5)	Ref	
1 wave	176 (13.2)	1.218 (0.992, 1.496)	0.059	143 (25.8)	1.329 (1.081, 1.633)	0.007	90 (23.3)	1.464 (1.131, 1.894)	0.004	209 (13.4)	1.317 (1.108, 1.565)	0.002
2 waves	139 (16.5)	1.586 (1.268, 1.983)	< 0.001	52 (28.3)	1.508 (1.083, 2.099)	0.015	24 (16.9)	0.982 (0.626, 1.543)	0.939	130 (16.3)	1.656 (1.343, 2.042)	< 0.001
3 waves	154 (23.5)	2.457 (1.966, 3.069)	< 0.001	31 (30.1)	1.648 (1.074, 2.530)	0.022	18 (30.0)	2.070 (1.180, 3.631)	0.011	72 (16.4)	1.669 (1.275, 2.184)	< 0.001
**Low muscle strength**												
0 wave	537 (13.2)	Ref		815 (20.6)	Ref		444 (17.0)	Ref		809 (11.5)	Ref	
1 wave	130 (16.6)	1.304 (1.058,	0.013	112 (32.0)	1.811 (1.428,	< 0.001	66 (22.2)	1.395 (1.042,	0.025	67 (20.2)	1.950 (1.477,	< 0.001

		1.607)			2.296)			1.868)			2.575)	
2 waves	45 (17.6)	1.404 (1.005, 1.962)	0.047	30 (28.0)	1.499 (0.976, 2.302)	0.064	26 (26.0)	1.716 (1.085, 2.713)	0.021	22 (21.2)	2.062 (1.281, 3.320)	0.003
3 waves	12 (35.3)	3.573 (1.758, 7.262)	< 0.001	18 (36.0)	2.164 (1.209, 3.876)	0.009	20 (38.5)	3.052 (1.729, 5.385)	< 0.001	11 (22.0)	2.168 (1.106, 4.249)	0.024
**Unhealthy weight status**												
0 wave	300 (11.7)	Ref		124 (20.8)	Ref		87 (14.5)	Ref		178 (8.4)	Ref	
1 wave	92 (14.6)	1.293 (1.005, 1.663)	0.045	60 (20.1)	0.958 (0.678, 1.352)	0.806	29 (14.3)	0.979 (0.622, 1.542)	0.927	77 (11.1)	1.363 (1.028, 1.808)	0.031
2 waves	83 (15.7)	1.408 (1.082, 1.833)	0.011	95 (25.4)	1.299 (0.957, 1.763)	0.093	49 (16.9)	1.194 (0.815, 1.750)	0.363	102 (12.5)	1.572 (1.215, 2.035)	0.001
3waves	249 (17.6)	1.613 (1.344, 1.935)	< 0.001	696 (21.8)	1.065 (0.859, 1.321)	0.563	391 (19.8)	1.454 (1.130, 1.875)	0.004	552 (14.3)	1.822 (1.525, 2.177)	< 0.001

Abbreviations: CHARLS, China Health and Retirement Longitudinal Study; HRS, Health and Retirement Study; ELSA, English Longitudinal Study of Ageing; SHARE, Survey of Health, Ageing and Retirement in Europe; No. (%), Number (percentage); OR, odds ratio; CI: confidence interval; Ref, reference group.

Table S18 Association between cumulative multi-system health status and CVD odds (without multiple interpolations)

	CHARLS			HRS			ELSA			SHARE		

	No. (%)	OR (95%CI)	P value	No. (%)	OR (95%CI)	P value	No. (%)	OR (95%CI)	P value	No. (%)	OR (95%CI)	P value
**Cognitive function impairment**												
0 wave	592 (14.1)	Ref		825 (22.0)	Ref		451 (17.5)	Ref		814 (11.8)	Ref	
1 wave	76 (13.7)	0.891 (0.683, 1.161)	0.392	85 (21.3)	0.889 (0.682, 1.160)	0.387	72 (20.2)	0.954 (0.691, 1.317)	0.774	75 (16.2)	1.287 (0.982, 1.688)	0.068
2 waves	41 (16.8)	1.121 (0.780, 1.609)	0.537	42 (22.5)	0.884 (0.610, 1.281)	0.515	25 (24.3)	1.362 (0.802, 2.313)	0.252	15 (14.6)	1.027 (0.579, 1.820)	0.928
3 waves	15 (11.2)	0.748 (0.429, `1.303)	0.305	23 (19.7)	0.708 (0.434, 1.154)	0.166	8 (34.8)	2.073 (0.798, 5.383)	0.134	5 (15.2)	1.008 (0.377, 2.692)	0.988
**Depression**												
0 wave	255 (11.1)	Ref		749 (20.7)	Ref		424 (17.2)	Ref		498 (10.5)	Ref	
1 wave	176 (13.2)	1.167 (0.946, 1.441)	0.150	143 (25.8)	1.393 (1.123, 1.729)	0.003	90 (23.3)	1,508 (1.117, 2.037)	0.007	209 (13.4)	1.378 (1.150, 1.651)	0.001
2 waves	139 (16.5)	1.507 (1.197, 1.898)	< 0.001	52 (28.3)	1.563 (1.106, 2.207)	0.011	24 (16.9)	1.246 (0.745, 2.084)	0.401	130 (16.3)	1.815 (1.454, 2.266)	< 0.001
3 waves	154 (23.5)	2.284 (1.809, 2.884)	< 0.001	31 (30.1)	2.010 (1.282, 3.152)	0.002	18 (30.0)	2.595 (1.331, 5.062)	0.005	72 (16.4)	1.780 (1.337, 2.369)	< 0.001

**Low muscle strength**												
0 wave	537 (13.2)	Ref		815 (20.6)	Ref		444 (17.0)	Ref		809 (11.5)	Ref	
1 wave	130 (16.6)	1.128 (0.906, 1.405)	0.280	112 (32.0)	1.368 (1.065, 1.757)	0.014	66 (22.2)	1.121 (0.803, 1.566)	0.503	67 (20.2)	1.261 (0.941, 1.689)	0.121
2 waves	45 (17.6)	1.089 (0.764, 1.554)	0.637	30 (28.0)	1.037 (0.665, 1.615)	0.874	26 (26.0)	1.320 (0.774, 2.252)	0.308	22 (21.2)	1.012 (0.614, 1.670)	0.961
3 waves	12 (35.3)	2.587 (1.235, 5.416)	0.012	18 (36.0)	1.601 (0.876, 2.924)	0.126	20 (38.5)	2.316 (1.194, 4.492)	0.013	11 (22.0)	1.141 (0.566, 2.301)	0.713
**Unhealthy weight status**												
0 wave	300 (11.7)	Ref		124 (20.8)	Ref		87 (14.5)	Ref		178 (8.4)	Ref	
1 wave	92 (14.6)	1.270 (0.982, 1.643)	0.069	60 (20.1)	0.888 (0.622, 1.269)	0.515	29 (14.3)	0.891 (0.540, 1.472)	0.653	77 (11.1)	1.204 (0.900, 1.610)	0.212
2 waves	83 (15.7)	1.275 (0.973, 1.672)	0.079	95 (25.4)	1.245 (0.908, 1.706)	0.173	49 (16.9)	1.187 (0.784, 1.797)	0.419	102 (12.5)	1.512 (1.158, 1.974)	0.002
3waves	249 (17.6)	1.399 (1.154, 1.695)	0.001	696 (21.8)	0.945 (0.753, 1.185)	0.623	391 (19.8)	1.265 (0.953, 1.678)	0.103	552 (14.3)	1.591 (1.316, 1.923)	< 0.001

Adjusted for age, gender, education level, marital status, hypertension, diabetes, drinking status, and smoking status.

Abbreviations: CHARLS, China Health and Retirement Longitudinal Study; HRS, Health and Retirement Study; ELSA, English Longitudinal Study of Ageing;

SHARE, Survey of Health, Ageing and Retirement in Europe; No. (%), Number (percentage); OR, odds ratio; CI: confidence interval; Ref, reference group.

Table S19 Association between cumulative multi-system health status and CVD odds (after multiple interpolations)

	CHARLS			HRS			ELSA			SHARE		
	No. (%)	OR (95%CI)	P value	No. (%)	OR (95%CI)	P value	No. (%)	OR (95%CI)	P value	No. (%)	OR (95%CI)	P value
**Cognitive function impairment**												
0 wave	592 (14.1)	Ref		825 (22.0)	Ref		451 (17.5)	Ref		814 (11.8)	Ref	
1 wave	76 (13.7)	0.896 (0.693, 1.160)	0.419	85 (21.3)	0.885 (0.680, 1.153)	0.366	72 (20.2)	1.061 (0.796, 1.416)	0.685	75 (16.2)	1.288 (0.982, 1.479)	0.066
2 waves	41 (16.8)	1.114 (0.776, 1.599)	0.558	42 (22.5)	0.884 (0.614, 1.273)	0.514	25 (24.3)	1.288 (0.801, 2.071)	0.296	15 (14.6)	1.024 (0.765, 1.372)	0.934
3 waves	15 (11.2)	0.750 (0.565, 1.307)	0.309	23 (19.7)	0.699 (0.430, 1.137)	0.151	8 (34.8)	1.986 (0.816, 4.832)	0.131	5 (15.2)	1.008 (0.610, 1.664)	0.988
**Depression**												
0 wave	255 (11.1)	Ref		749 (20.7)	Ref		424 (17.2)	Ref		498 (10.5)	Ref	
1 wave	176	1.183	0.117	143	1.399	0.002	90	1.592	0.001	209	1.362	0.001

	(13.2)	(0.959, 1.458)		(25.8)	(1.129, 1.734)		(23.3)	(1.219, 2.080)		(13.4)	(1.180, 1.573)	
2 waves	139 (16.5)	1.495 (1.329, 1.681)	0.001	52 (28.3)	1.540 (1.091, 2.174)	0.014	24 (16.9)	1.104 (0.694, 1.757)	0.676	130 (16.3)	1.804 (1.612, 2.020)	< 0.001
3 waves	154 (23.5)	2.277 (1.805, 2.871)	< 0.001	31 (30.1)	1.984 (1.343, 2.930)	0.003	18 (30.0)	2.250 (1.260, 4.018)	0.006	72 (16.4)	1.771 (1.531, 2.050)	< 0.001
**Low muscle strength**												
0 wave	537 (13.2)	Ref		815 (20.6)	Ref		444 (17.0)	Ref		809 (11.5)	Ref	
1 wave	130 (16.6)	1.126 (0.905, 1.259)	0.287	112 (32.0)	1.391 (1.070, 1.809)	0.009	66 (22.2)	1.196 (0.884, 1.617)	0.248	67 (20.2)	1.013 (0.785, 1.308)	0.960
2 waves	45 (17.6)	1.085 (0.905, 1.300)	0.652	30 (28.0)	1.036 (0.826, 1.298)	0.877	26 (26.0)	1.418 (0.884, 2.274)	0.149	22 (21.2)	1.013 (0.785, 1.308)	0.960
3 waves	12 (35.3)	2.631 (1.804, 3.835)	0.010	18 (36.0)	1.599 (0.875, 2.174)	0.127	20 (38.5)	2.346 (1.303, 4.226)	0.004	11 (22.0)	1.113 (0.554, 2.238)	0.763
**Unhealthy weight status**												
0 wave	300 (11.7)	Ref		124 (20.8)	Ref		87 (14.5)	Ref		178 (8.4)	Ref	

1 wave	92 (14.6)	1.269 (0.892, 1.563)	0.068	60 (20.1)	0.906 (0.639, 1.284)	0.582	29 (14.3)	0.913 (0.577, 1.444)	0.696	77 (11.1)	1.206 (0.901, 1.398)	0.207
2 waves	83 (15.7)	1.275 (0.983, 1.653)	0.077	95 (25.4)	1.238 (0.904, 1.453)	0.184	49 (16.9)	1.077 (0.731, 1.586)	0.708	102 (12.5)	1.512 (1.160, 1.972)	0.002
3waves	249 (17.6)	1.391 (1.148, 1.684)	0.001	696 (21.8)	0.944 (0.753, 1.184)	0.620	391 (19.8)	1.300 (1.001, 1.689)	0.049	552 (14.3)	1.601 (1.328, 1.931)	< 0.001

Adjusted for age, gender, education level, marital status, hypertension, diabetes, drinking status, and smoking status.

Abbreviations: CHARLS, China Health and Retirement Longitudinal Study; HRS, Health and Retirement Study; ELSA, English Longitudinal Study of Ageing; SHARE, Survey of Health, Ageing and Retirement in Europe; No. (%), Number (percentage); OR, odds ratio; CI: confidence interval; Ref, reference group.

Table S20 subgroup analysis of cumulative unhealthy system number and CVD odds (after multiple interpolations)

	CHARLS (n=5127)		HRS (n=4458)		ELSA (n=3061)		SHARE (n=7511)	
	OR (95%CI)	P for interacton	OR (95%CI)	P for interacton	OR (95%CI)	P for interacton	OR (95%CI)	P for interacton
Age		0.105		0.289		0.169		0.038
< 65	1.363 (1.212, 1.533)***		1.222 (1.021, 1.462)*		1.343 (1.128, 1.601)**		1.574 (1.354, 1.830)***	
≥ 65	1.217 (1.005, 1.474)*		1.136 (0.983, 1.312)		1.263 (1.039, 1.536)*		1.367 (1.205, 1.550)***	
Sex		0.305		0.792		0.184		0.926
Male	1.405 (1.195,		1.239 (1.027,		1.194 (0.987,		1.477 (1.283,	

	1.652)***		1.495)*		1.444)		1.701)***	
Female	1.264 (1.113, 1.436)***		1.130 (0.983, 1.300)		1.369 (1.149, 1.633)**		1.442 (1.262, 1.647)***	
Education level		0.816		0.162		0.681		0.177
Below high school	1.323 (1.192, 1.468)***		1.068 (0.818, 1.393)		1.520 (1.196, 1.932)**		1.404 (1.234, 1.597)***	
High school	1.447 (0.985, 2.126)		1.267 (1.096, 1.465)**		1.095 (0.916, 1.310)		1.282 (1.057, 1.555)*	
Above high school	0.550 (0.084, 3.615)		1.113 (0.868, 1.427)		1.615 (1.182, 2.206)**		1.853 (1.479, 1.231)***	
Marital status		0.195		0.047		0.204		0.543
Married	1.347 (1.211, 1.499)***		1.217 (1.058, 1.401)**		1.348 (1.149, 1.581)***		1.420 (1.267, 1.592)***	
Others	1.164 (0.870, 1.558)		1.096 (0.908, 1.323)		1.191 (0.956, 1485)		1.531 (1.278, 1.836)***	
Now smoking		0.990		0.093		0.171		0.990
No	1.320 (1.175, 1.484)***		1.122 (0.994, 1.267)		1.249 (1.089, 1.433)**		1.439 (1.294, 1.599)***	
Yes	1.292 (1.064, 1.570)*		1.456 (1.083, 1.959)*		1.643 (1.109, 2.434)*		1.478 (1.165, 1.874)**	
Now drinking		0.565		0.138		0.899		0.248
No	1.357 (1.207, 1.527)***		1.110 (0.945, 1.304)		1.324 (0.895, 1.957)		1.543 (1.332, 1.787)***	
Yes	1.222 (1.008, 1.483)*		1.219 (1.042, 1.427)*		1.277 (1.114, 1.464)***		1.379 (1.212, 1.569)***	
Hypertension		0.022		0.439		0.651		< 0.001

No	1.411 (1.251, 1.591)***		1.112 (0.941, 1.313)		1.249 (1.061, 1.470)**		1.299 (1.142, 1.478)***	
Yes	1.123 (0.938, 1.344)		1.215 (1.044, 11.415)*		1.356 (1.097, 1.675)**		1.570 (1.350, 1.826)***	
Diabetes		0.899		0.094		0.845		0.012
No	1.320 (1.190, 1.465)***		1.196 (1.058, 1.352)**		1.304 (1.142, 1.489)***		1.450 (1.307, 1.608)***	
Yes	1.426 (0.961, 2.114)		1.058 (0.804, 1.391)		1.062 (0.608, 1.857)		1.282 (0.960, 1.713)	

Adjusted for age, gender, education level, marital status, hypertension, diabetes, drinking status, and smoking status.

Abbreviations: CHARLS, China Health and Retirement Longitudinal Study; HRS, Health and Retirement Study; ELSA, English Longitudinal Study of Ageing; SHARE, Survey of Health, Ageing and Retirement in Europe; OR, odds ratio; CI: confidence interval; Ref, reference group.

Table S21 Association between cumulative multi-system health status and CVD odds (excluded underweight after multiple interpolations)

	**CHARLS**			**HRS**			**ELSA**			**SHARE**		
	No. (%)	OR (95%CI)	P value	No. (%)	OR (95%CI)	P value	No. (%)	OR (95%CI)	P value	No. (%)	OR (95%CI)	P value
**Unhealthy system number**												
0 system in 3 waves	73 (12.8)	Ref		75 (19.1)	Ref		55 (14.1)	Ref		90 (7.1)	Ref	
1 system in 1 of the 3 waves	283 (12.8)	1.492 (1.136, 1.960)	0.005	543 (20.1)	0.929 (0.807, 1.071)	0.606	285 (16.5)	1.090 (0.793, 1.497)	0.596	447 (11.3)	1.511 (1.258, 1.816)	0.001

2 systems in 1 of the 3 waves	256 (18.1)	1.999 (1.500, 2.665)	< 0.001	285 (25.3)	1.163 (0.874, 1.546)	0.326	163 (21.6)	1.444 (1.025, 2.035)	0.036	298 (15.4)	2.047 (1.582, 2.649)	< 0.001
3/4 systems in 1 of the 3 waves	77 (22.3)	2.385 (1.648, 3.453)	< 0.001	66 (29.7)	1.397 (0.981, 1.990)	0.108	52 (29.7)	2.073 (1.318, 3.261)	0.002	68 (23.0)	2.822 (1.965, 4.052)	< 0.001

Adjusted for age, gender, education level, marital status, hypertension, diabetes, drinking status, and smoking status.

Abbreviations: CHARLS, China Health and Retirement Longitudinal Study; HRS, Health and Retirement Study; ELSA, English Longitudinal Study of Ageing; SHARE, Survey of Health, Ageing and Retirement in Europe; No. (%), Number (percentage); OR, odds ratio; CI: confidence interval; Ref, reference group.

Table S22 Adherence to JoGH's GRABDROP guidelines items

JoGH guideline items
1. Please list all papers published by each co-author in previous 3 years that were based on secondary analysis of a big data repository
Xin Y, Qian X, Yang Y. Association between systolic blood pressure trajectories and successful aging in middle-aged and elderly Chinese individuals: a longitudinal study. BMC Public Health. 2025 Dec 30;25(1):4390.
Xin Y, Qian X, Yang Y. Associations between sarcopenic obesity and the risk of cardiovascular-kidney-metabolic syndrome progression: insights from the China health and retirement longitudinal study. Diabetol Metab Syndr. 2025 Dec 29;17(1):460.
Xin Y, Qian X, Yang Y. Association between C-reactive protein-triglyceride-glucose index and the risk of cardiovascular-kidney-metabolic syndrome progression: insights from the China Health and

Retirement Longitudinal Study. Nutr Metab Cardiovasc Dis. 2026 Apr 1:104725.<br><br>Xin Y, Lyu S, Wang J, Wang Y, Shu Y, Liang H, Yang Y. Association between social risk profile, cardiovascular risk factors control, and future cardiovascular risk. BMC Public Health. 2025 Jul 3;25(1):2340.<br><br>Xin Y, Wang Y, Shu Y, Liang H, Yang Y. Correlation between conventional and unconventional lipid parameters with the risk of progression of renal function decline: insights from the China Health and Retirement Longitudinal Study 2011-2015. Ren Fail. 2025 Dec;47(1):2499229.<br><br>Xin Y, Wang Y, Shu Y, Liang H, Yang Y. Association between remnant cholesterol and low-density lipoprotein cholesterol discordance and type 2 diabetes or prediabetes: results from NHANES. BMC Endocr Disord. 2025 Jul 7;25(1):168.	
2. Please explain the key elements of your study design and the use of the available datasets that make your study an original scientific contribution	
This study conducted a longitudinal multi-cohort analysis using four large publicly available aging cohorts (CHARLS, ELSA, HRS, SHARE). While each dataset has been used in prior studies, this study uniquely integrates multi-system trajectories (cognitive, psychological, physical function, BMI) and examines their associations with incident cardiovascular disease across cohorts, providing a novel multi-domain perspective.	
3. Please list all publications that addressed similar research questions in the same dataset and indicate where you cited them in your paper	
Wu S, Yang X, Chen Y, et al. Changes in depressive symptoms as predictors of incident cardiovascular disease: insights from four prospective cohorts. Eur J Prev Cardiol. 2025; Published online. doi:10.1093/eurjpc/zwaf586 (2.3 Outcome Ref. 27)	

Zeng Q, Zhao L, Zhong Q, An Z, Li S. Changes in sarcopenia and incident cardiovascular disease in prospective cohorts. BMC Med. 2024;22(1):607. doi:10.1186/s12916-024-03841-x (2.3 Outcome Ref. 28)

Prasitsiriphon O, Pothisiri W. Associations of Grip Strength and Change in Grip Strength With All-Cause and Cardiovascular Mortality in a European Older Population. *Clin Med Insights: Cardiol*. 2018;12:1179546818771894. doi:10.1177/1179546818771894 (4 Discussion Ref. 31)

Wu Q, Xu J, Xu X, Yang J. Trajectories of depressive symptoms and the risk of cardiovascular, dementia, and pulmonary events in older adults: Evidence from the English Longitudinal Study of Ageing. *Gen Hosp Psychiatry*. 2025;94:199-205 (4 Discussion Ref. 33)

4. Please explain how you addressed multiple testing through an appropriately rigorous statistical threshold and indicate this in the methods section

The primary analyses were based on pre-specified hypotheses examining the associations between multi-system health status, multi-system health trajectories, and incident cardiovascular disease. Therefore, no formal adjustment for multiple testing was applied. Subgroup and interaction analyses were conducted as secondary/exploratory analyses to assess the consistency of findings across population subgroups.

5. Please declare to what extent have AI chatbots been used in developing your paper and to which parts of the paper did they contribute

AI chatbots (e.g., ChatGPT) were used solely for language editing and improving clarity in certain sections of the manuscript (e.g., refining phrasing in Methods and Discussion). All scientific content, data analysis, and interpretation were performed exclusively by the authors.

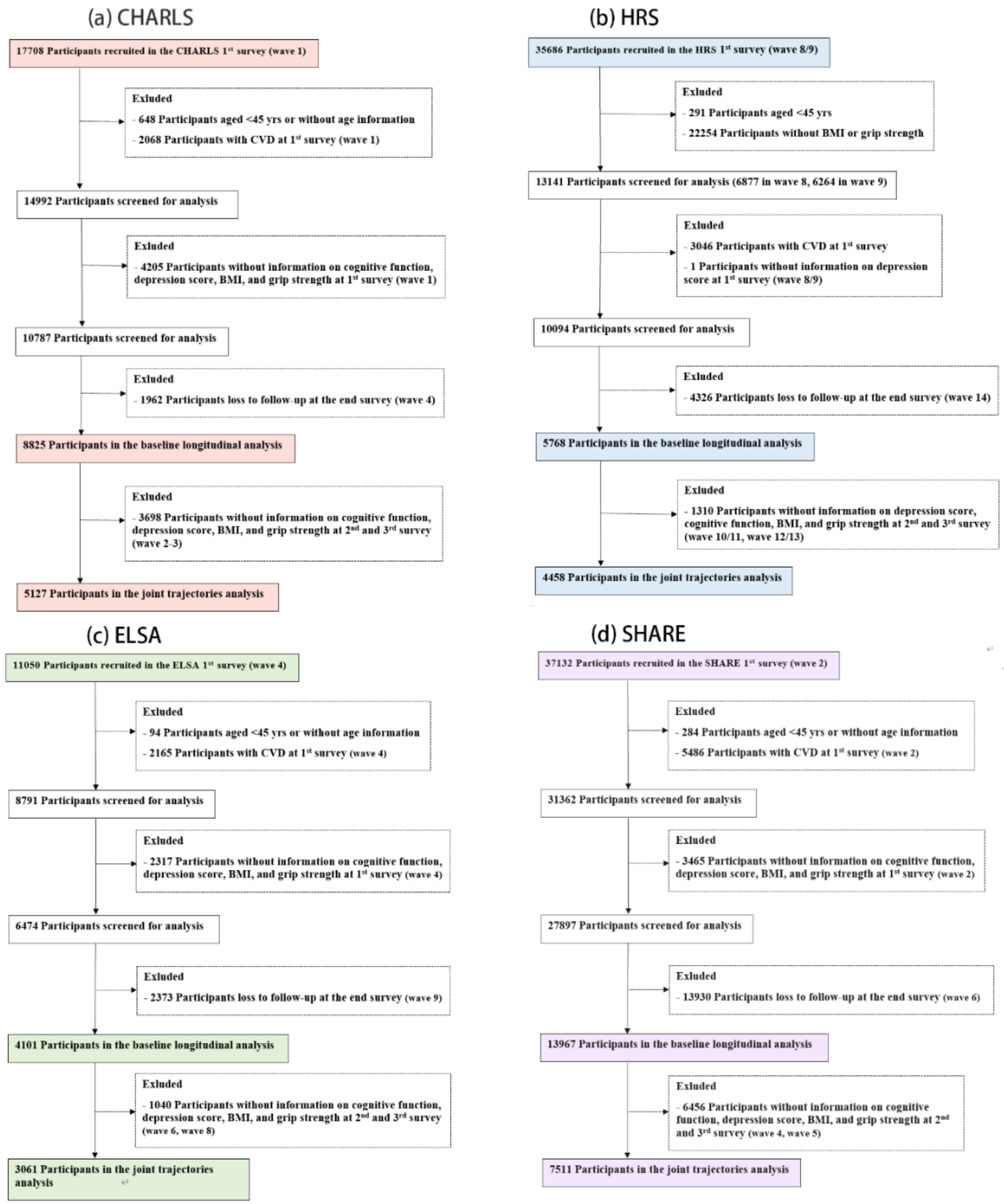
(a) CHARLS
17708 Participants recruited in the CHARLS 1st survey (wave 1)
Exluded
- 648 Participants aged <45 yrs or without age information
- 2068 Participants with CVD at 1st survey (wave 1)
14992 Participants screened for analysis
Exluded
- 4205 Participants without information on cognitive function, depression score, BMI, and grip strength at 1st survey (wave 1)
10787 Participants screened for analysis
Exluded
- 1962 Participants loss to follow-up at the end survey (wave 4)
8825 Participants in the baseline longitudinal analysis
Exluded
- 3698 Participants without information on cognitive function, depression score, BMI, and grip strength at 2nd and 3rd survey (wave 2-3)
5127 Participants in the joint trajectories analysis
(b) HRS
35686 Participants recruited in the HRS 1st survey (wave 8/9)
Exluded
- 291 Participants aged <45 yrs
- 22254 Participants without BMI or grip strength
13141 Participants screened for analysis (6877 in wave 8, 6264 in wave 9)
Exluded
- 3046 Participants with CVD at 1st survey
- 1 Participants without information on depression score at 1st survey (wave 8/9)
10094 Participants screened for analysis
Exluded
- 4326 Participants loss to follow-up at the end survey (wave 14)
5768 Participants in the baseline longitudinal analysis
Exluded
- 1310 Participants without information on depression score, cognitive function, BMI, and grip strength at 2nd and 3rd survey (wave 10/11, wave 12/13)
4458 Participants in the joint trajectories analysis
(c) ELSA
11050 Participants recruited in the ELSA 1st survey (wave 4)
Exluded
- 94 Participants aged <45 yrs or without age information
- 2165 Participants with CVD at 1st survey (wave 4)
8791 Participants screened for analysis
Exluded
- 2317 Participants without information on cognitive function, depression score, BMI, and grip strength at 1st survey (wave 4)
6474 Participants screened for analysis
Exluded
- 2373 Participants loss to follow-up at the end survey (wave 9)
4101 Participants in the baseline longitudinal analysis
Exluded
- 1040 Participants without information on cognitive function, depression score, BMI, and grip strength at 2nd and 3rd survey (wave 6, wave 8)
3061 Participants in the joint trajectories analysis
(d) SHARE
37132 Participants recruited in the SHARE 1st survey (wave 2)
Exluded
- 284 Participants aged <45 yrs or without age information
- 5486 Participants with CVD at 1st survey (wave 2)
31362 Participants screened for analysis
Exluded
- 3465 Participants without information on cognitive function, depression score, BMI, and grip strength at 1st survey (wave 2)
27897 Participants screened for analysis
Exluded
- 13930 Participants loss to follow-up at the end survey (wave 6)
13967 Participants in the baseline longitudinal analysis
Exluded
- 6456 Participants without information on cognitive function, depression score, BMI, and grip strength at 2nd and 3rd survey (wave 4, wave 5)
7511 Participants in the joint trajectories analysis

Figure S1 flow chart of the population selection process

Abbreviations: CHARLS, China Health and Retirement Longitudinal Study; HRS, Health and Retirement Study; ELSA, English Longitudinal Study of Ageing; SHARE, Survey of Health, Ageing and Retirement in Europe; CVD, Cardiovascular diseases; BMI, Body Mass Index

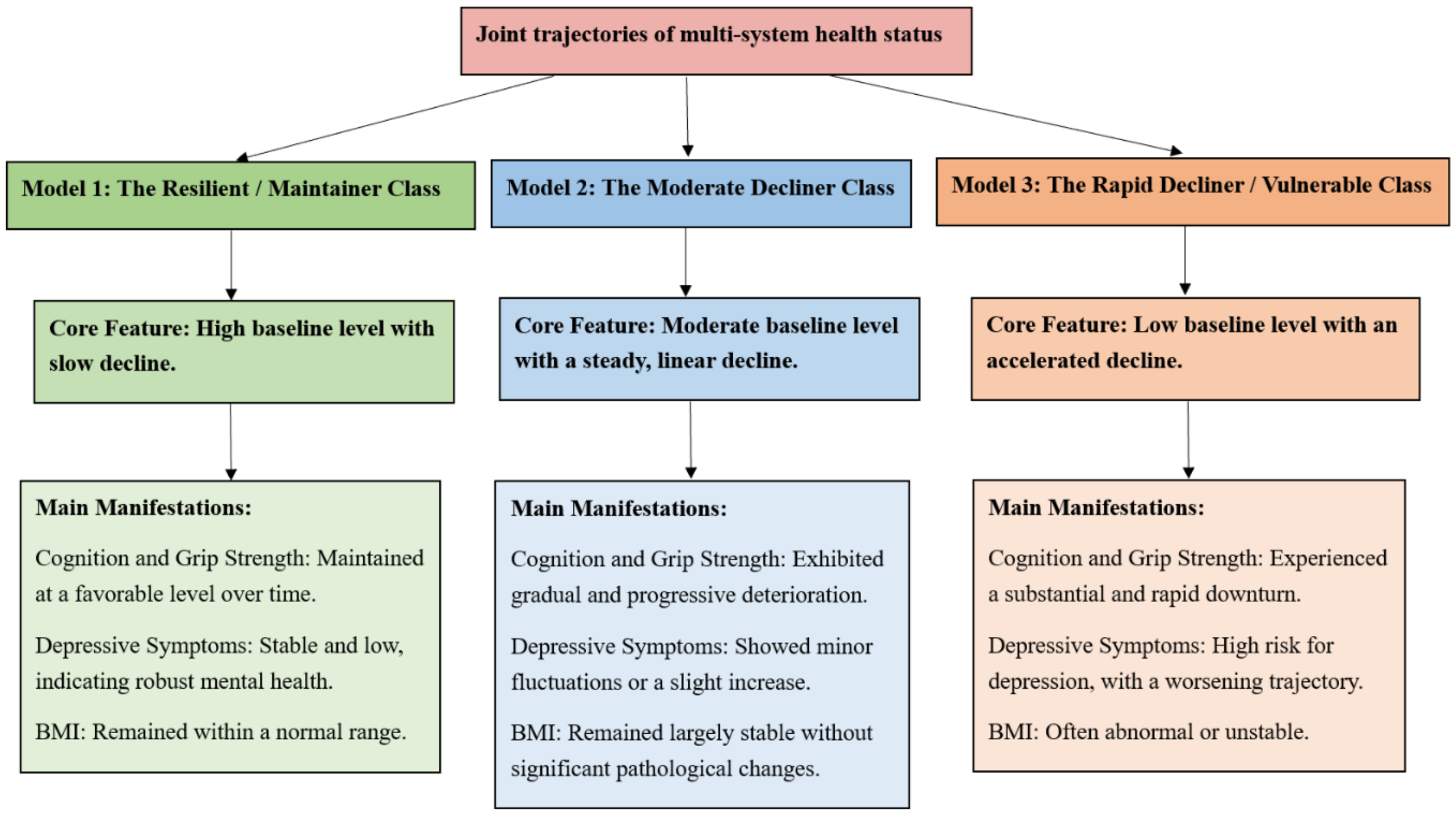

Figure S2 Multi-system health status trajectories models

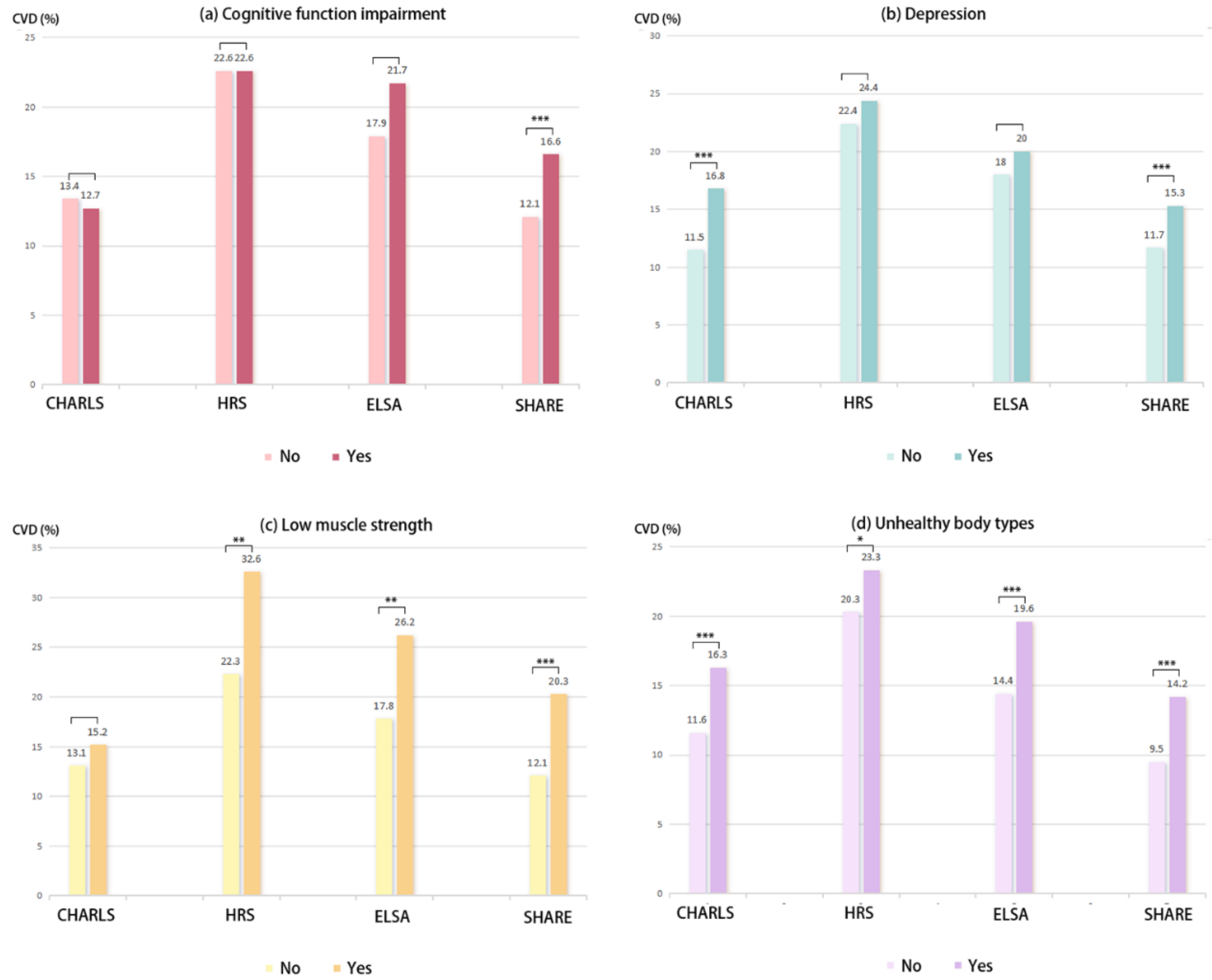

Figure S3 baseline multi-system health status and the incidence of CVD in the last wave

Abbreviations: CHARLS, China Health and Retirement Longitudinal Study; HRS, Health and Retirement Study; ELSA, English Longitudinal Study of Ageing; SHARE, Survey of Health, Ageing and Retirement in Europe.

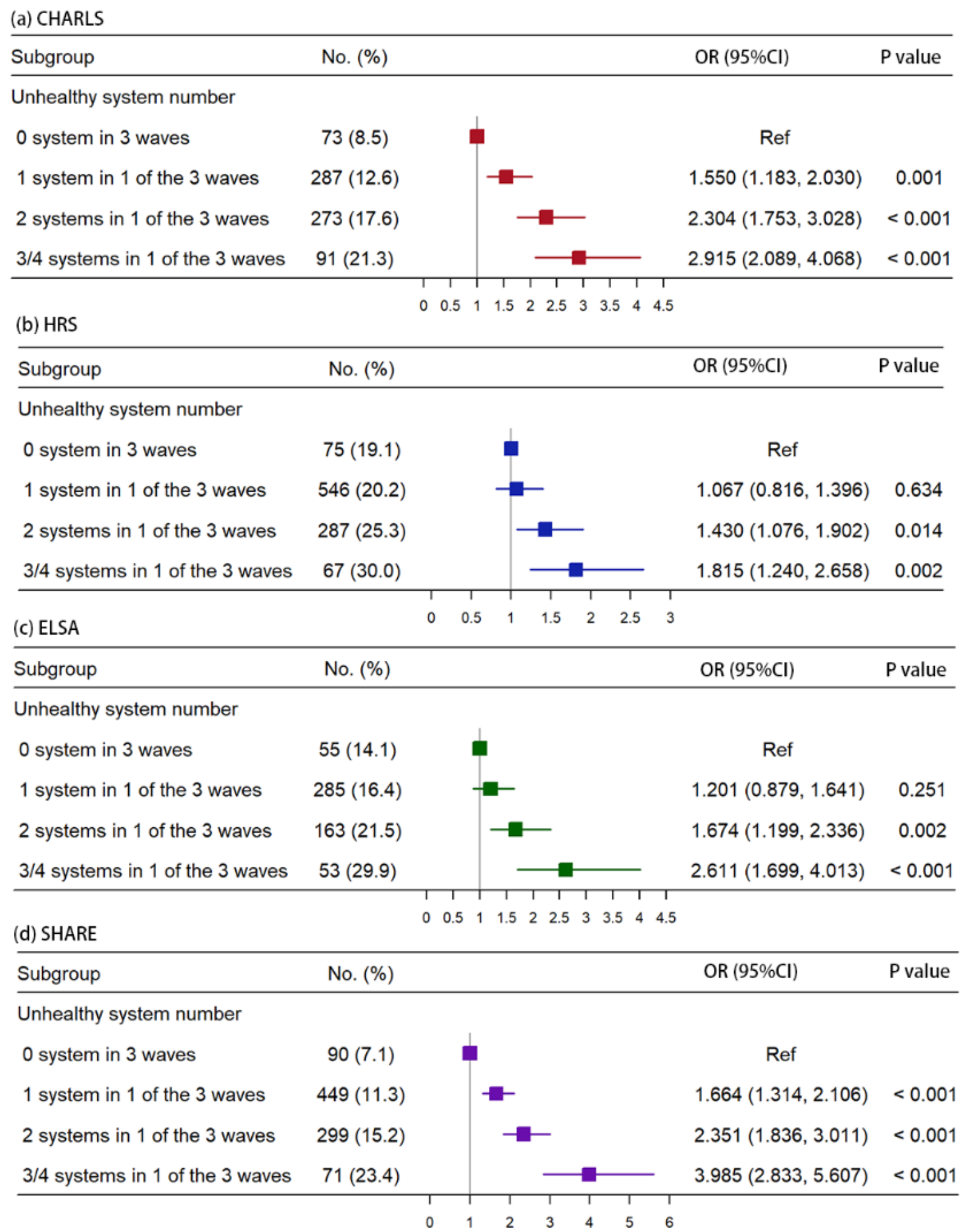

Figure S4 Association between cumulative multi-system health status and CVD odds (unadjusted)

Abbreviations: CHARLS, China Health and Retirement Longitudinal Study; HRS, Health and Retirement Study; ELSA, English Longitudinal Study of Ageing; SHARE, Survey of Health, Ageing and Retirement in Europe; No. (%), Number (percentage); OR, odds ratio; CI: confidence interval; Ref, reference group.

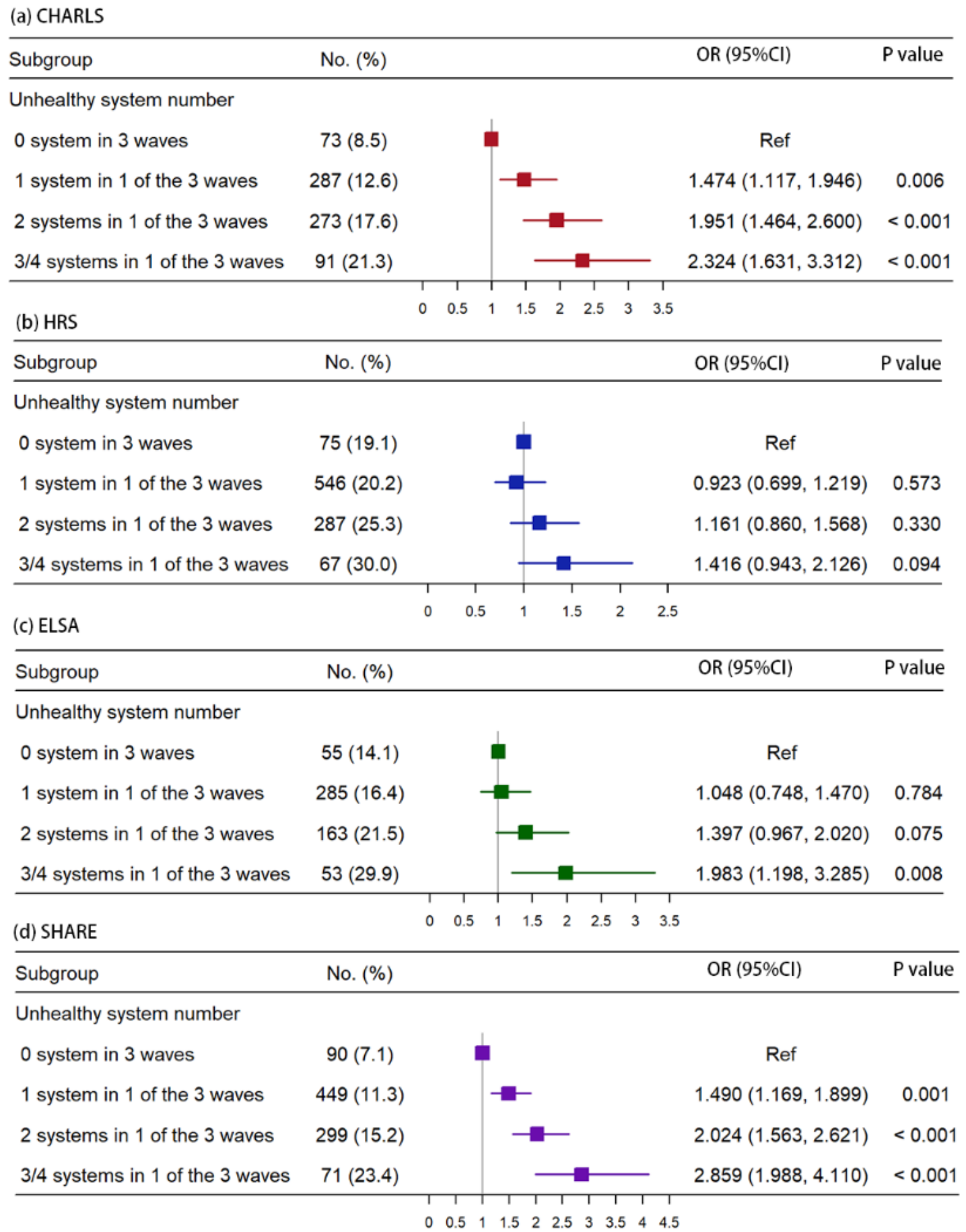

Figure S5 Association between cumulative multi-system health status and CVD ods (without multiple interpolations)

Adjusted for age, gender, education level, marital status, hypertension, diabetes, drinking status, and smoking status.

Abbreviations: CHARLS, China Health and Retirement Longitudinal Study; HRS, Health and Retirement Study; ELSA, English Longitudinal Study of Ageing; SHARE, Survey of Health, Ageing and Retirement in Europe; No. (%), Number (percentage); OR, odds ratio; CI: confidence interval; Ref, reference group.

Supplementary Methods1: Multi-system health status assessment

Cognitive function was assessed using a composite score derived from various cognitive tasks (Table S3). Impairment was defined as a score below the age-group-specific mean minus 1.5 standard deviations (Table S1). Participants were grouped into five age categories for these calculations: ≥45 to <55, ≥55 to <65, ≥65 to <75, 75 to <85, and ≥85 years.

Psychological condition was assessed using validated depression scales (Table S4). Specifically, the 10-item Center for Epidemiologic Studies Depression Scale (CESD-10) was utilized in CHARLS, the 8-item Center for Epidemiologic Studies Depression Scale (CES-D-8) in HRS and ELSA, and the 12-item EURO-D scale in SHARE. Psychological impairment was defined by depression, identified using specific cut-off scores for each scale: a score of 10 or higher for CESD-10[4], and a score of 4 or higher for both CES-D-8 and EURO-D[5] (Table S4).

Weight status were classified using Body Mass Index (BMI), derived from height and weight measurements (Table S5). Height and weight were directly measured in CHARLS, HRS, and ELSA, while self-reported in SHARE. For CHARLS participants, classification followed the Chinese criteria[6]: underweight (BMI < 18.5 kg/cm2), overweight (BMI ≥ 25 kg/cm2 and < 28 kg/cm2), and obesity (BMI ≥ 28 kg/cm2). Conversely, for ELSA, HRS, and SHARE cohorts, the European and American criteria were applied[7]: underweight (BMI < 18.5 kg/cm2), overweight (BMI ≥ 25 kg/cm2 and < 30 kg/cm2), and obesity (BMI ≥ 30 kg/cm2). Unhealthy weight status included underweight, overweight, and obesity categories (Table S1).

Muscle strength was assessed in all cohorts (CHARLS, HRS, ELSA, SHARE) using the maximum grip strength measurement of the dominant hand. Low muscle strength was defined by specific, regionally-guided thresholds (Table S6). For CHARLS, this definition followed the 2019 Asian Working Group for Sarcopenia (AWGS) criteria[8], specifically grip strength below 28 kg for males and below 18 kg for females. For HRS and ELSA, low muscle strength was defined according to the 2019 European Working Group on Sarcopenia in Older People (EWGSOP) criteria[9], with thresholds of below 27 kg for males and

below 16 kg for females (Table S6)

1. Poole L, Jackowska M. The Epidemiology of Depressive Symptoms and Poor Sleep: Findings from the English Longitudinal Study of Ageing (ELSA). Int J Behav Med. 2018;25:151–61. https://doi.org/10.1007/s12529-017-9703-y.

2. Talaei M, Feng L, Barrenetxea J, Yuan J-M, Pan A, Koh W-P. Adiposity, Weight Change, and Risk of Cognitive Impairment: The Singapore Chinese Health Study. J Alzheimers Dis. 2020;74:319–29. https://doi.org/10.3233/JAD-191052.

3. Andresen EM, Malmgren JA, Carter WB, Patrick DL. Screening for depression in well older adults: evaluation of a short form of the CES-D (Center for Epidemiologic Studies Depression Scale). Am J Prev Med. 1994;10:77–84.

4. Obesity: preventing and managing the global epidemic. Report of a WHO consultation. World Health Organ Tech Rep Ser. 2000;894:i–xii, 1–253.

5. Chen L-K, Woo J, Assantachai P, Auyeung T-W, Chou M-Y, Iijima K, et al. Asian Working Group for Sarcopenia: 2019 Consensus Update on Sarcopenia Diagnosis and Treatment. J Am Med Dir Assoc. 2020;21:300-307.e2. https://doi.org/10.1016/j.jamda.2019.12.012.

6. Cruz-Jentoft AJ, Bahat G, Bauer J, Boirie Y, Bruyère O, Cederholm T, et al. Sarcopenia: revised European consensus on definition and diagnosis. Age Ageing. 2019;48:16–31. https://doi.org/10.1093/ageing/afy169.

Supplementary Methods2: Group-Based Multi-Trajectory Modeling

Group-Based Multi-Trajectory Modeling (GBMTM) was used to identify distinct longitudinal patterns across cognitive function, psychological condition, weight status, and grip strength. Models with two to four latent classes were estimated and compared. The optimal number of trajectory classes was determined by jointly considering model fit, classification quality, trajectory adequacy, and clinical interpretability. Model fit was evaluated using the Akaike Information Criterion (AIC), Bayesian Information Criterion (BIC), Consistent Akaike Information Criterion (CAIC), and Sample-Size Adjusted Bayesian Information Criterion (SSBIC), with lower values indicating better model fit. The elbow criterion was additionally used to identify the point beyond which improvements in fit became marginal. Classification quality was assessed using the Average Posterior Probability of Assignment (APPA), Odds of Correct Classification (OCC), and entropy R^2. Consistent with recommended standards, APPA values ≥0.70, OCC values ≥5, and entropy R^2 ≥0.80 were considered indicative of adequate classification quality. To ensure statistical stability and clinical relevance, each trajectory class was required to contain at least 5% of the study population. The final model was selected based on the balance of statistical fit, classification accuracy, adequate class size, and clinical interpretability.

Rationale for selecting the three-class solution.

Although information criteria continued to decrease as additional classes were added, the reduction became progressively smaller beyond the three-class model (elbow criterion). Moreover, the three-class solution demonstrated excellent classification performance across all cohorts (APPA > 0.92, OCC > 23, entropy > 0.87), adequate class sizes, and clinically distinct and interpretable trajectory patterns. Additional classes increased model complexity without yielding substantively meaningful new trajectory patterns. Therefore, the three-class solution was selected as the most parsimonious and clinically meaningful model.
